# Supplementary material for: Synthesis of 5-Benzylamino and 5-Alkylamino-Substituted Pyrimido[4,5-c]quinoline Derivatives as CSNK2A Inhibitors with Antiviral Activity
Source: Pharmaceuticals (Basel). 2024 Feb 27;17(3):306. doi: 10.3390/ph17030306 (PMC10976173; doi:10.3390/ph17030306)
Supplement: Supplementary file 1 [file pharmaceuticals-17-00306-s001.zip › pharmaceuticals-2881461-supplementary.pdf]

**Supplementary Information**

# Synthesis of 5-Benzylamino and 5-Alkylamino-substituted Pyrimido[4,5-c]quinoline Derivatives as CSNK2A Inhibitors with Antiviral Activity

Kesatebrhan Haile Asressu[1], Jeffery L. Smith[1], Rebekah J. Dickmander[2,3,4,5], Nathaniel J. Moorman[2,3,4], James Wellnitz[6], Konstantin I. Popov[7], Alison D. Axtman[1,2], and Timothy M. Willson[1,2*]

[1] *Structural Genomics Consortium, UNC Eshelman School of Pharmacy, University of North Carolina at Chapel Hill, Chapel Hill, NC 27599, United States;* kesateha@unc.edu (K.H.A); jeffs@email.unc.edu (J.L.S)

[2] *Rapidly Emerging Antiviral Drug Development Initiative (READDI), Chapel Hill, NC 27599, United States*

[3] *Department of Microbiology & Immunology, University of North Carolina at Chapel Hill, Chapel Hill, NC 27599, United States;*

[4] *Lineberger Comprehensive Cancer Center, University of North Carolina at Chapel Hill, Chapel Hill, NC 27599, United States.*

[5] *Department of Chemistry, University of North Carolina at Chapel Hill, Chapel Hill, NC 27599, United States.*

[6] *Laboratory for Molecular Modeling, Division of Chemical Biology and Medicinal Chemistry, UNC Eshelman School of Pharmacy, University of North Carolina at Chapel Hill, Chapel Hill, NC 27599, United States*

[7] *Center of Integrative Chemical Biology and Drug Discovery, UNC Eshelman School of Pharmacy, University of North Carolina at Chapel Hill, Chapel Hill, NC 27599, United States*

* Correspondence: tim.willson@unc.edu

**Table of Contents** **Page**

Figure S1. $IC_{50}$ curves for CSNK2 NanoBRET assays S2–3

Figure S2. $IC_{50}$ curves for MHV-NLuc assays S4

Figures S3-S37 NMR spectra of all final compounds S5–39

**Figure S1.** $IC_{50}$ curves for CSNK2 NanoBRET assays

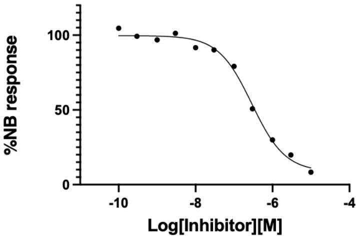


**4a**

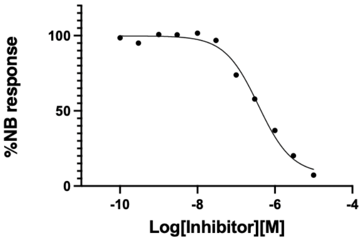


**4b**

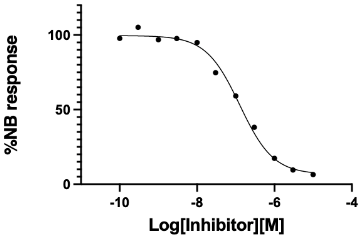


**4c**

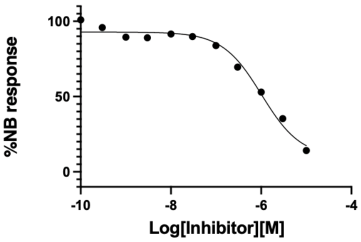


**4d**

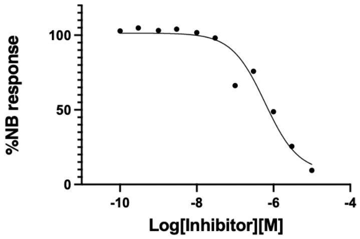


**4e**

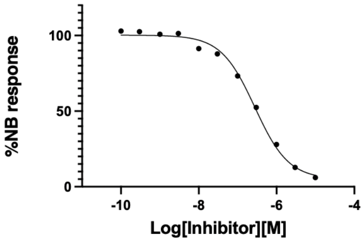


**4f**

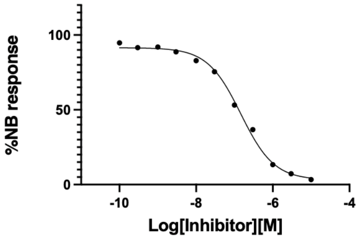


**4g**

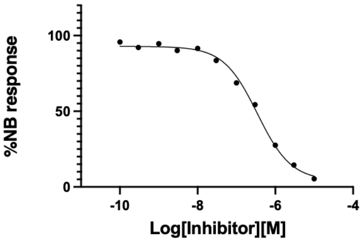


**4h**

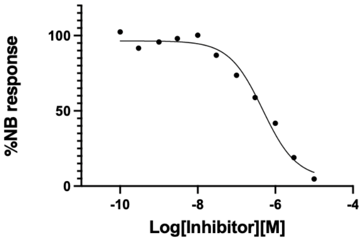


**4i**

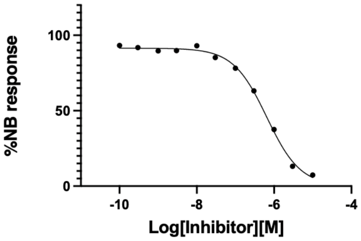


**4j**

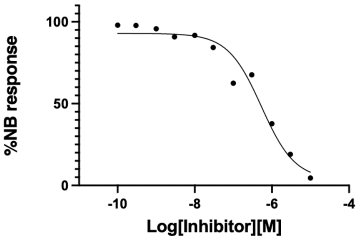


**4k**

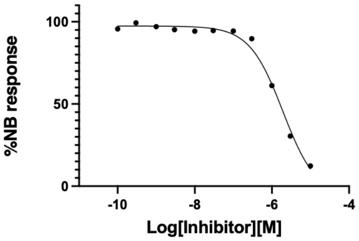


**4l**

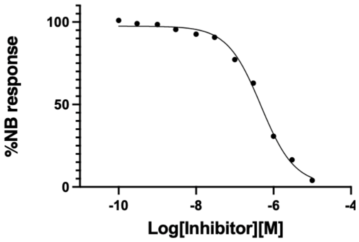


**4m**

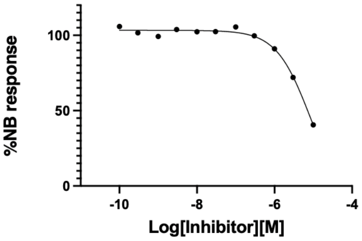


**4n**

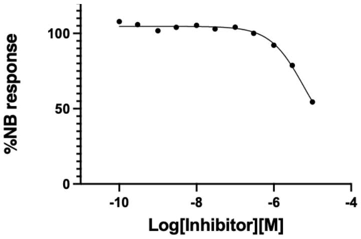


**4o**

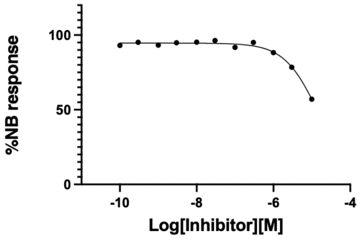


**4p**

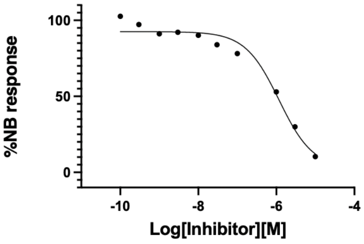


**4q**

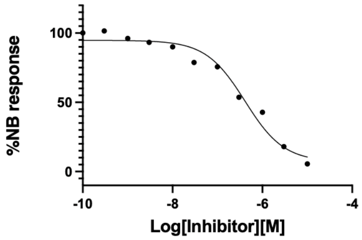


**4r**

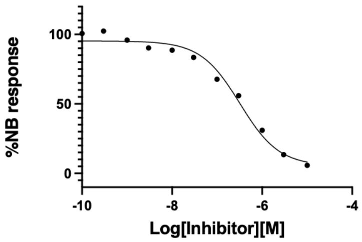


**4s**

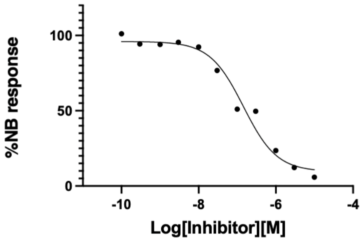


**4t**

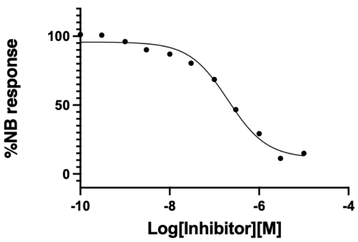


**4u**

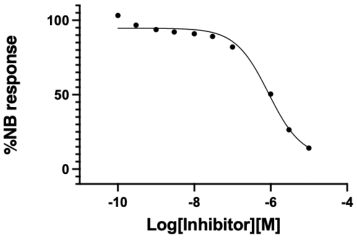


**4v**

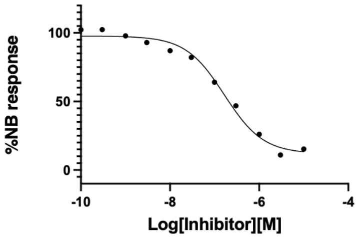


**4w**

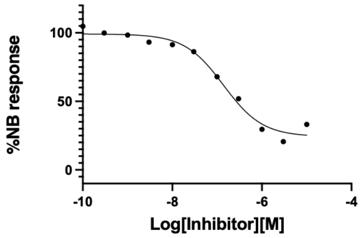


**4x**

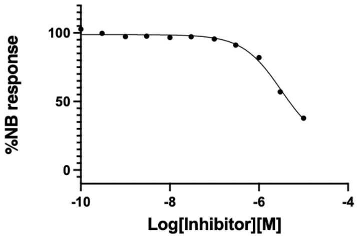


**4y**

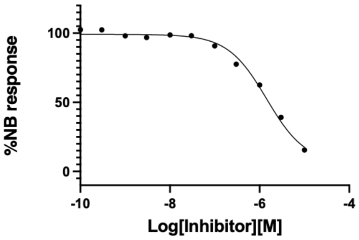


**4z**

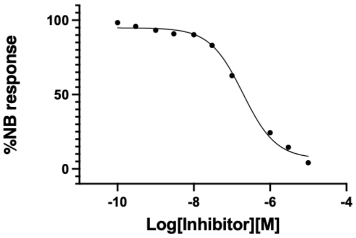


**4aa**

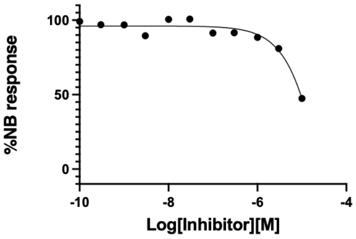


**4ab**

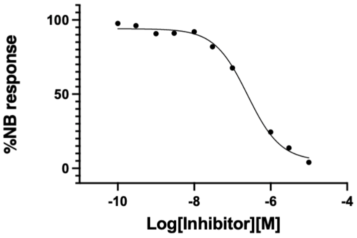


**4ac**

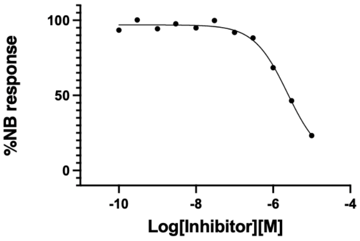


**4ad**

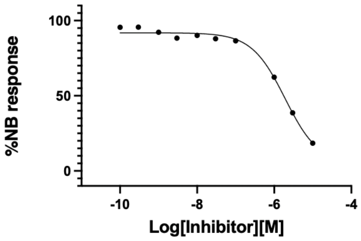


**4ae**

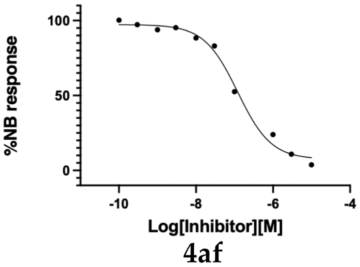


**4af**

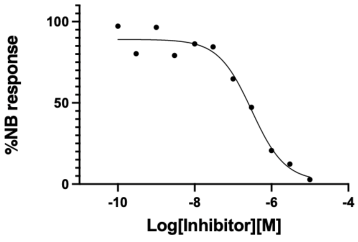


**4ag**

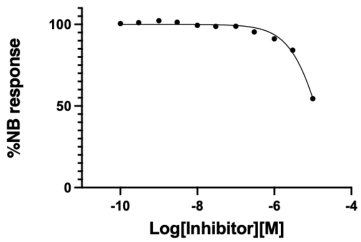


**4ah**

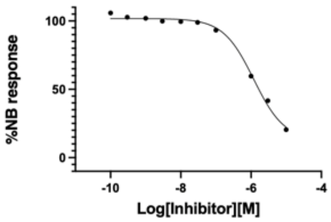


**4ai**

**Figure S2.** $IC_{50}$ curves for MHV-NLuc assays

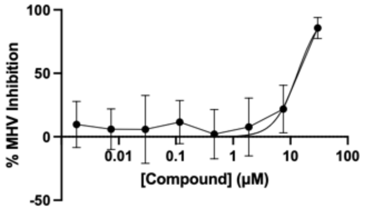


**4c**

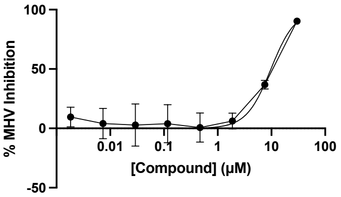


**4g**

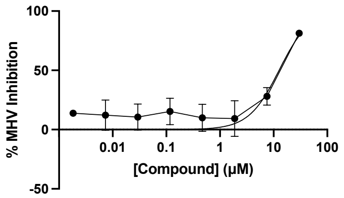


**4t**

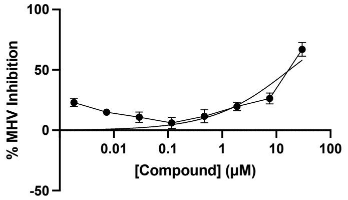


**4u**

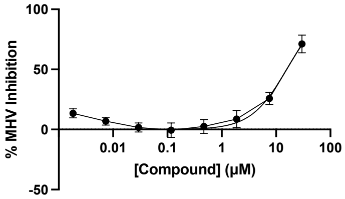


**4w**

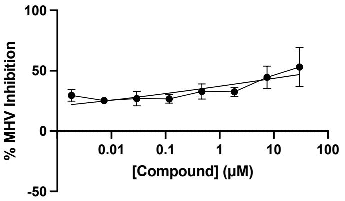


**4x**

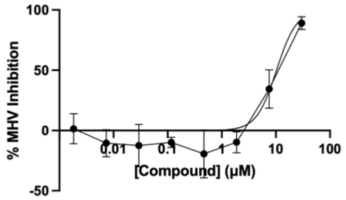


**4aa**

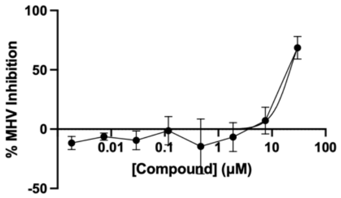


**4ag**

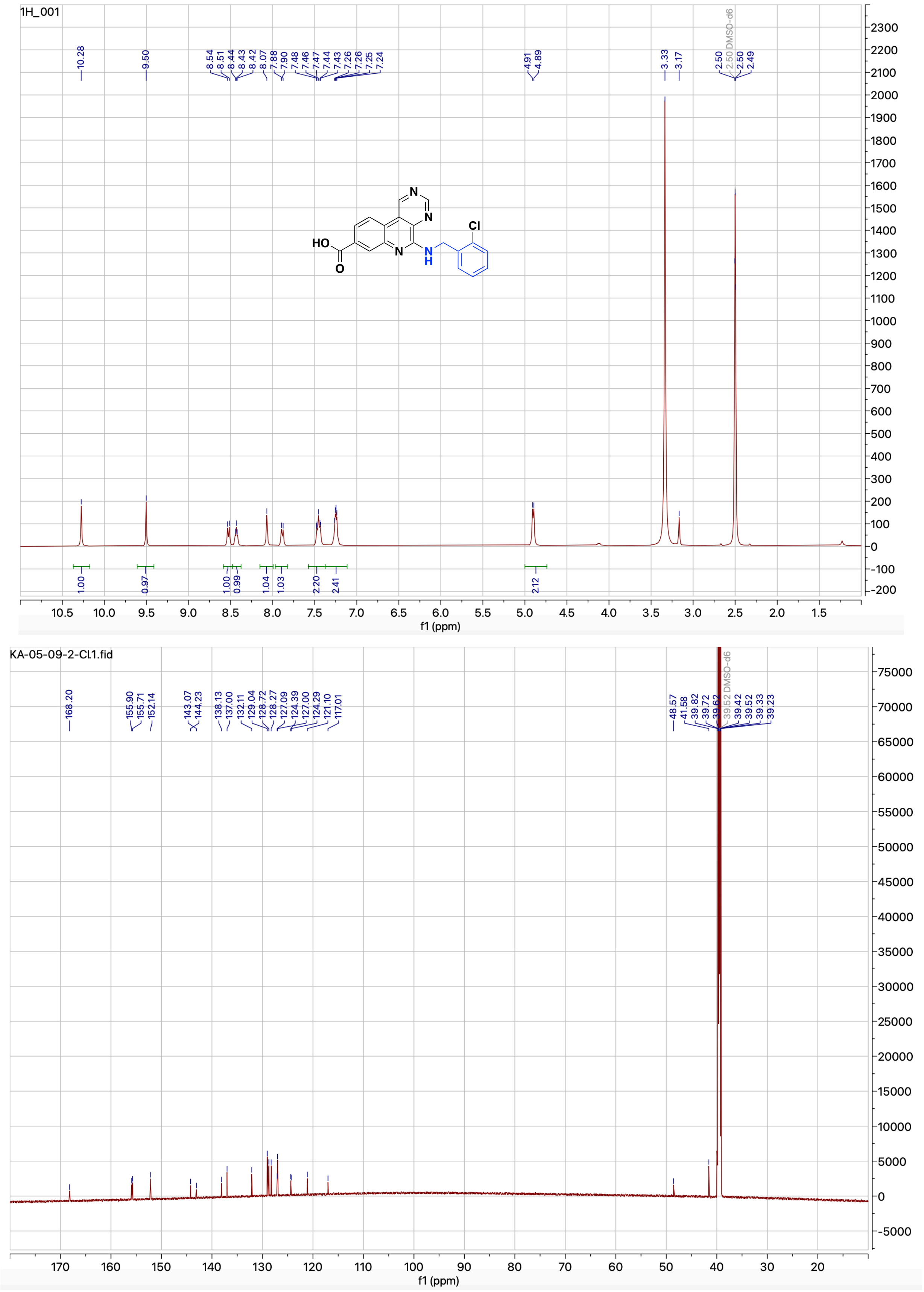


**Figure S3.** $^{1}H$ and $^{13}C$ NMR spectrum of compound **4a**

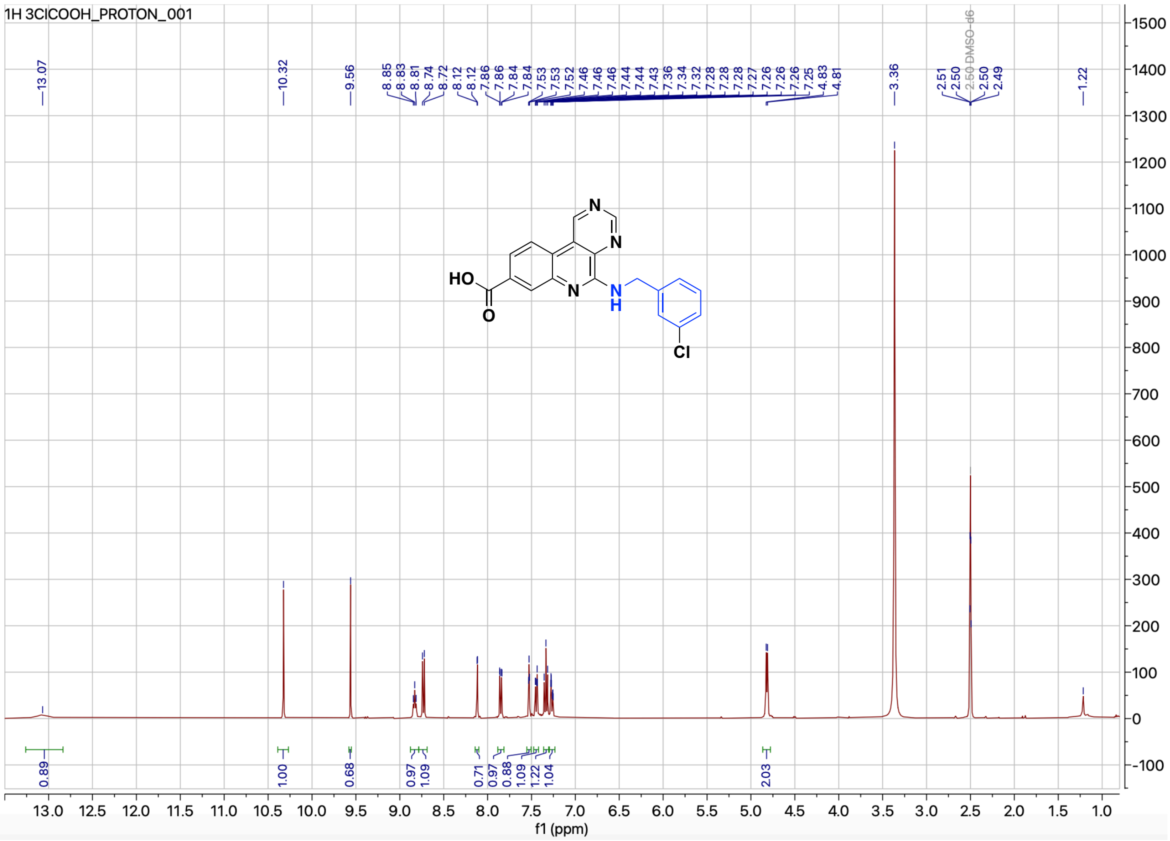


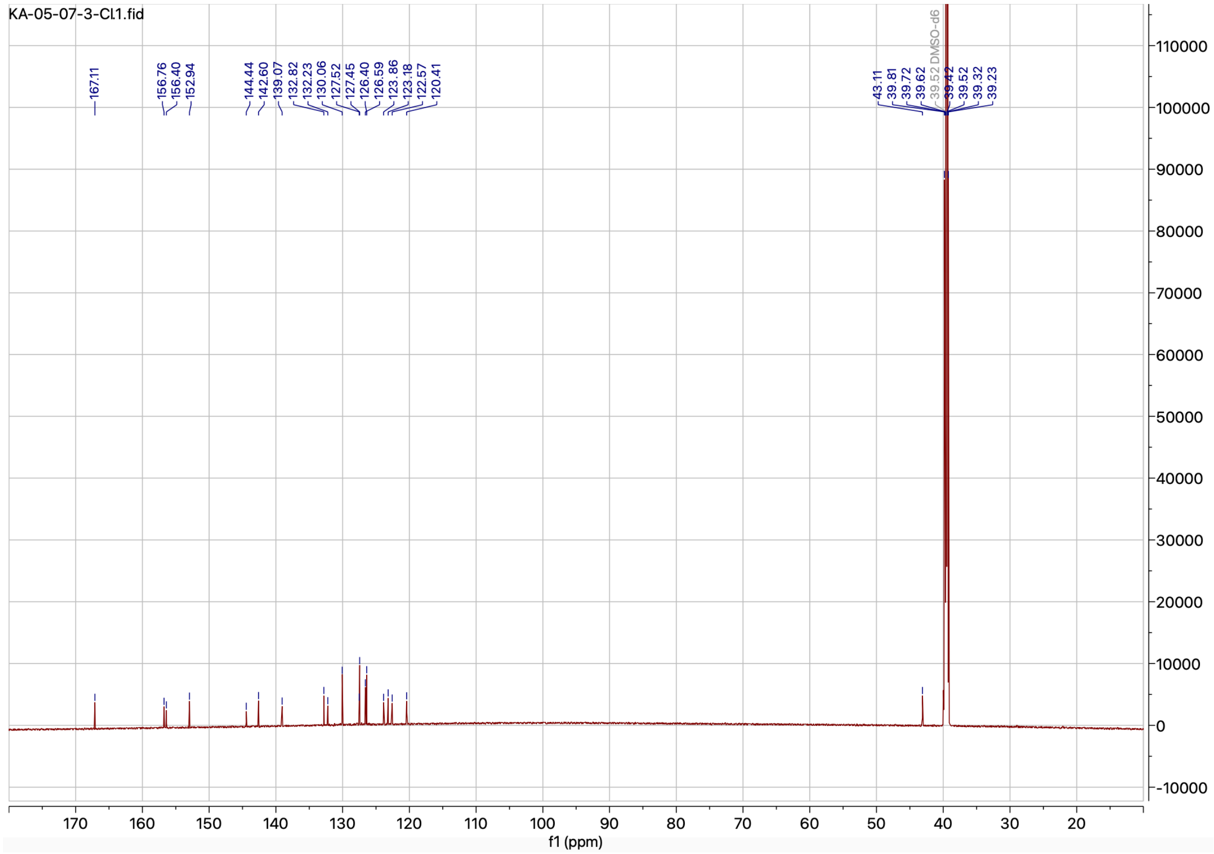


**Figure S4.** $^{1}$H and $^{13}$C NMR spectrum of compound **4b**

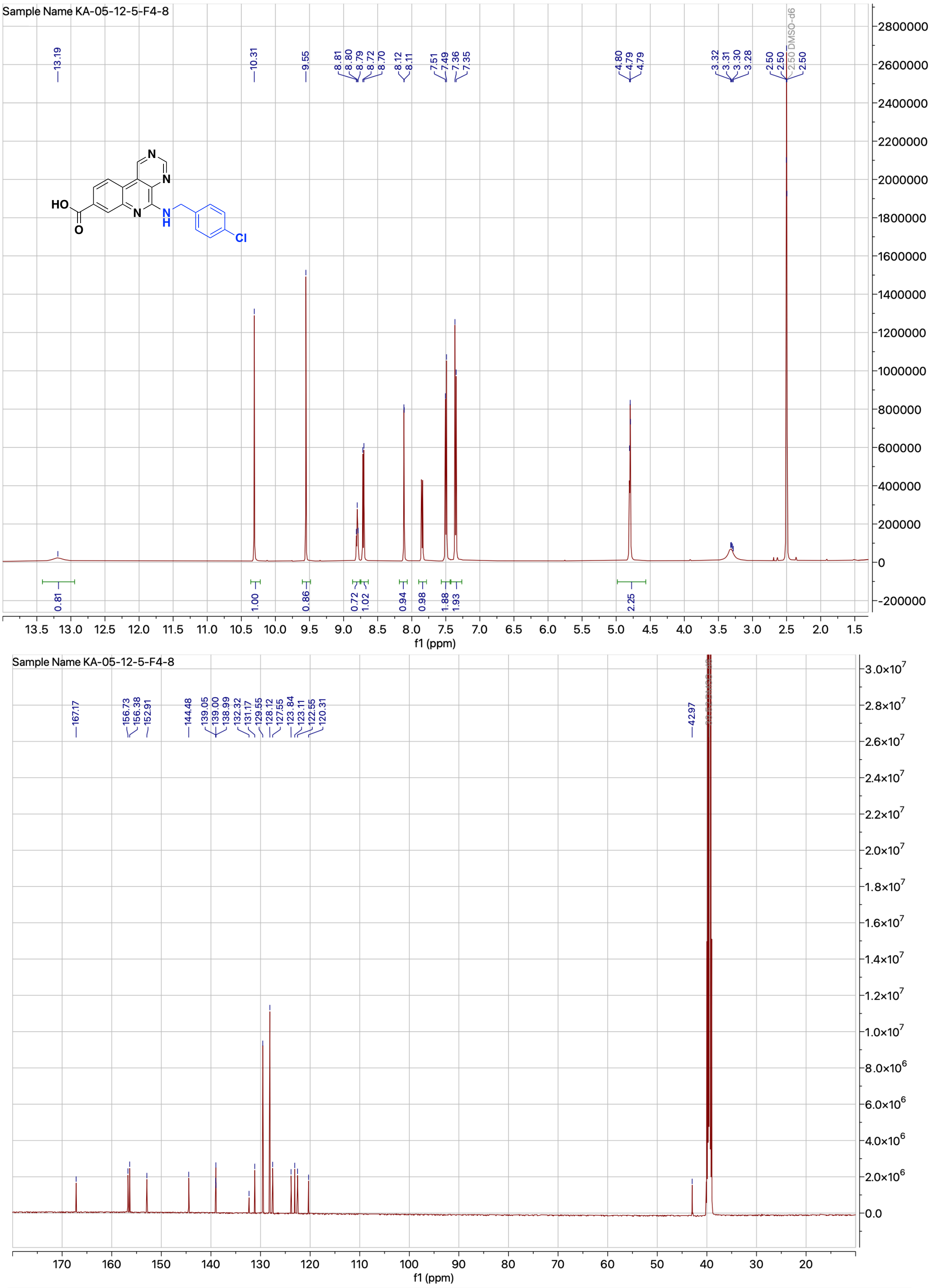


**Figure S5.** $^{1}$H and $^{13}$C NMR spectrum of compound **4c**

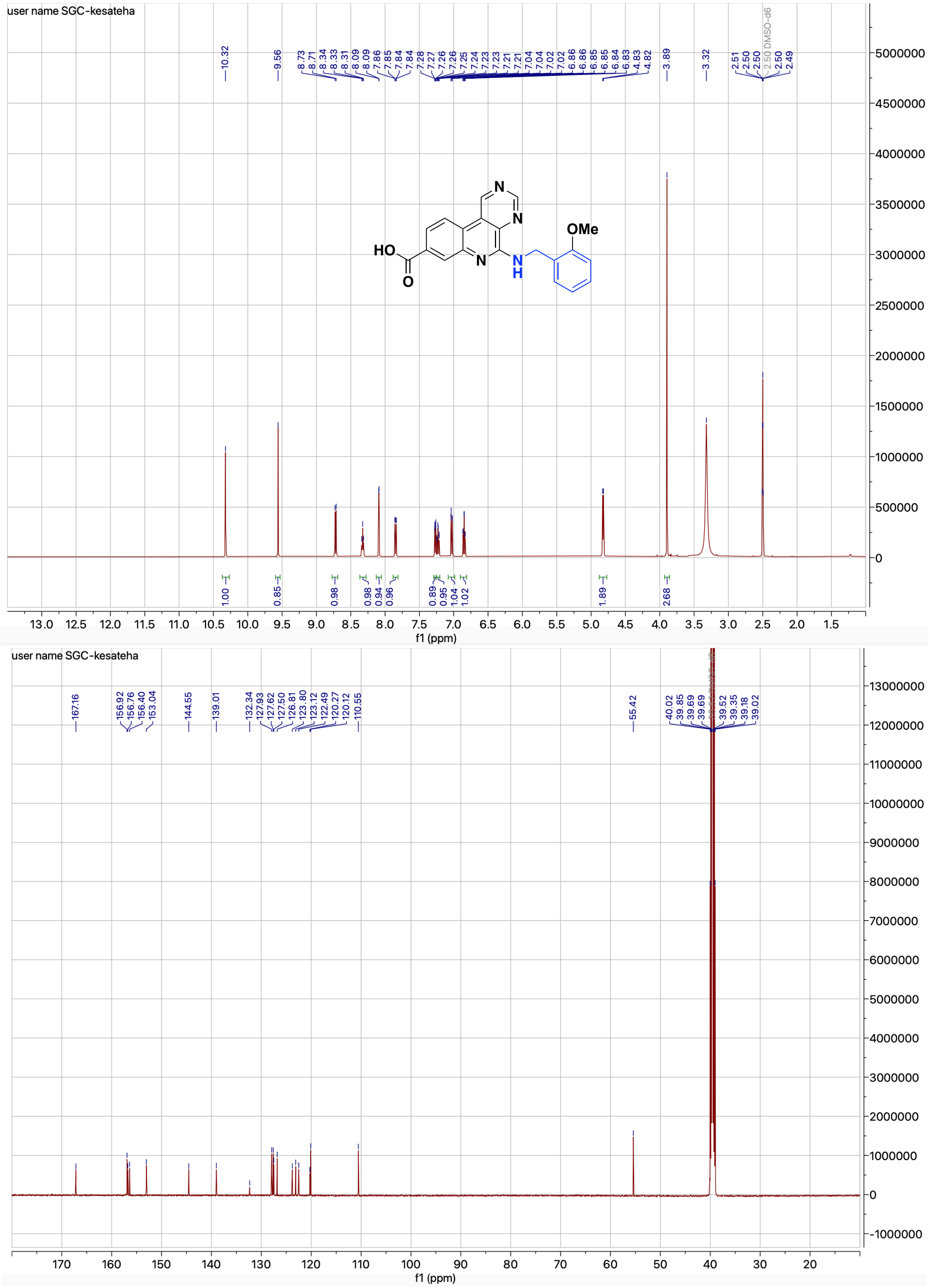

**Figure S6.** $^{1}H$ and $^{13}C$ NMR spectrum of compound **4d**

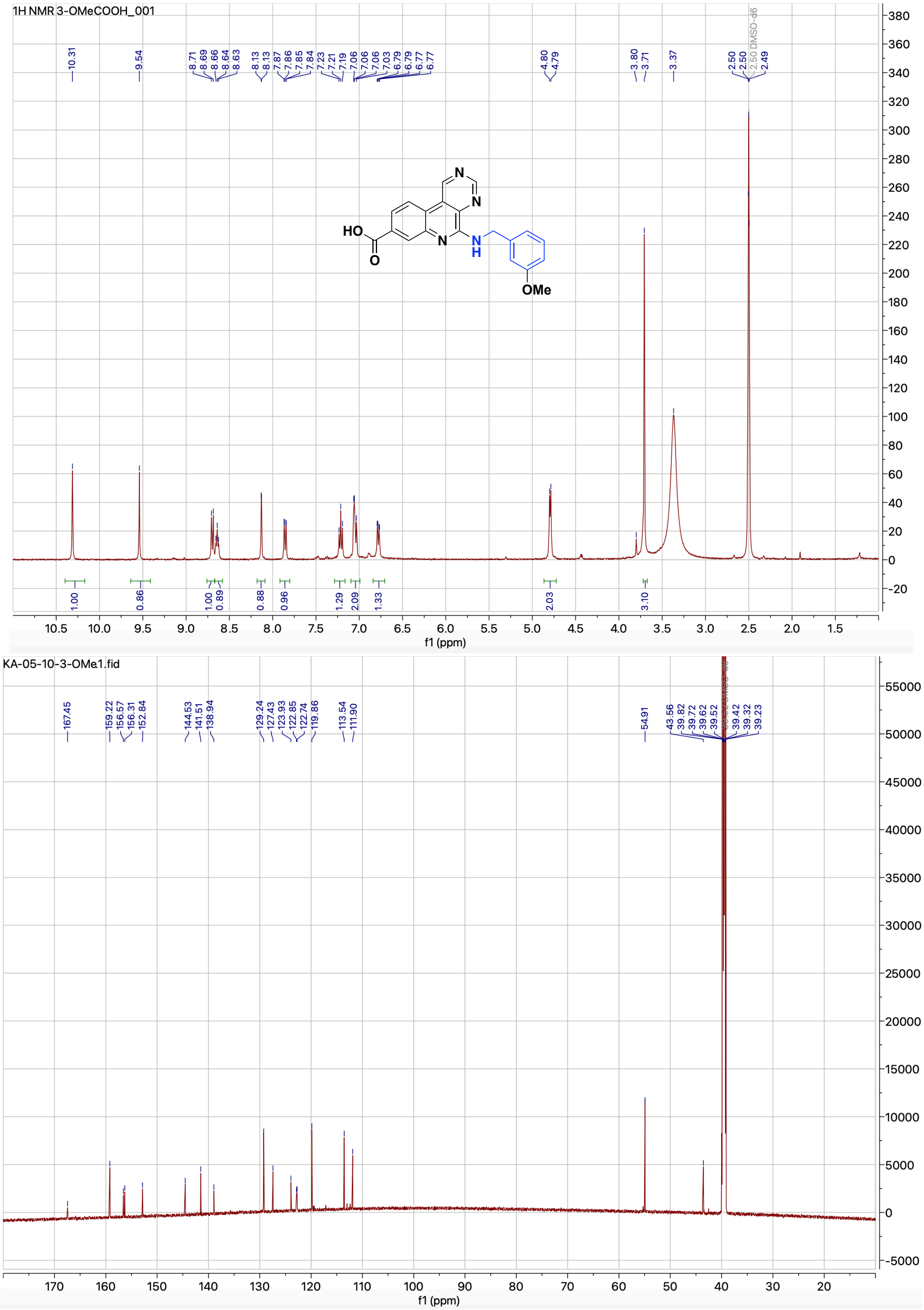


**Figure S7.** $^{1}$H and $^{13}$C NMR spectrum of compound **4e**

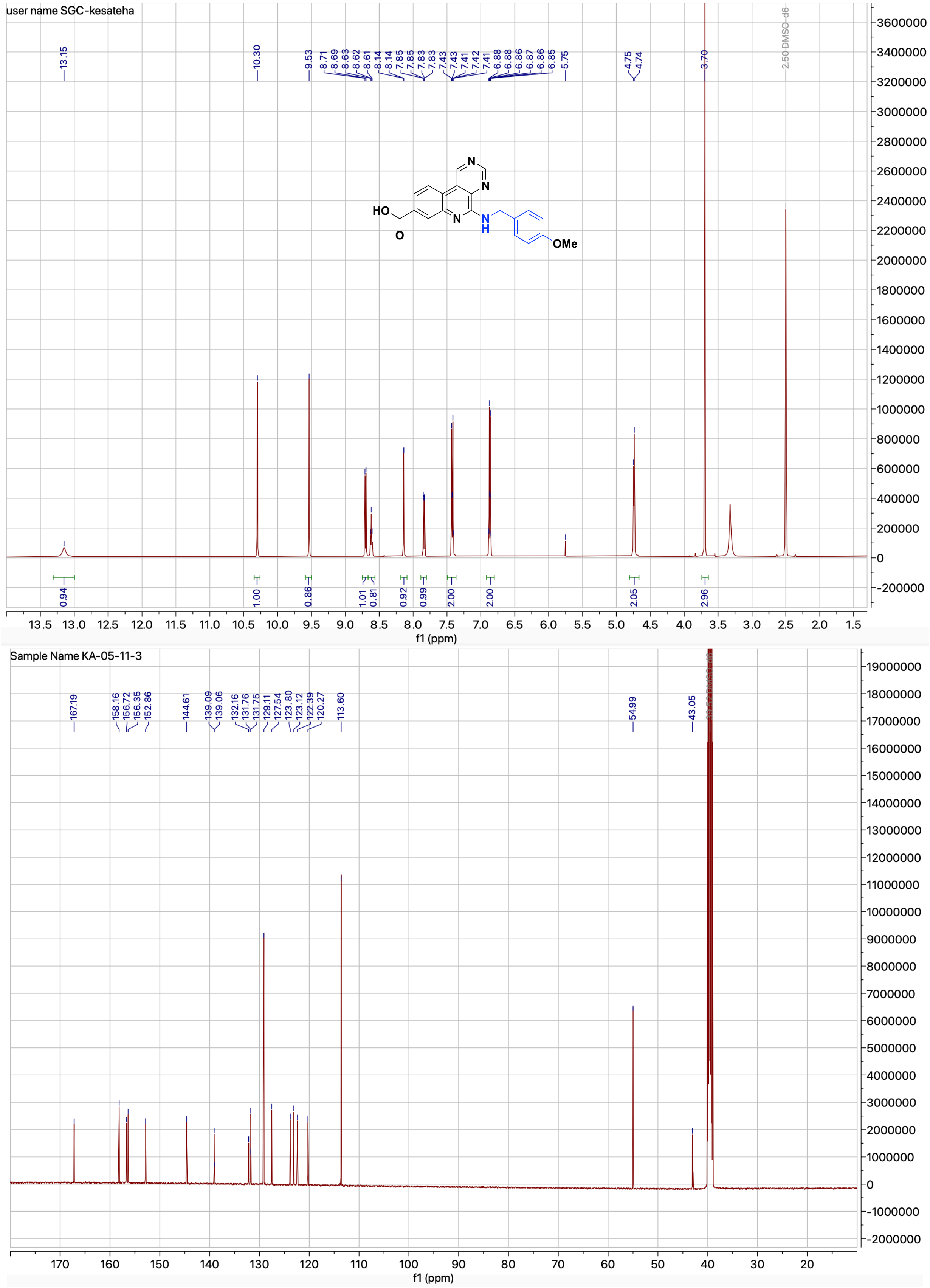


**Figure S8.** $^{1}$H and $^{13}$C NMR spectrum of compound **4f**

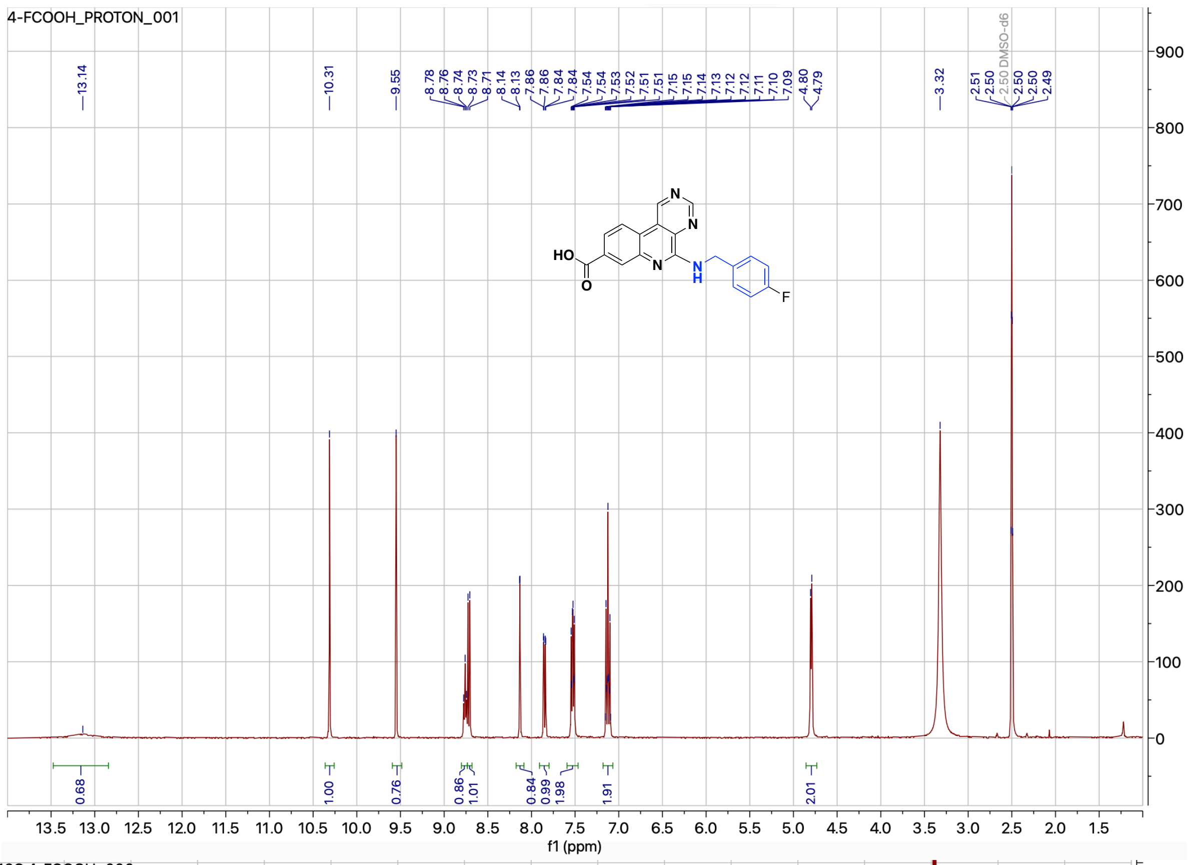

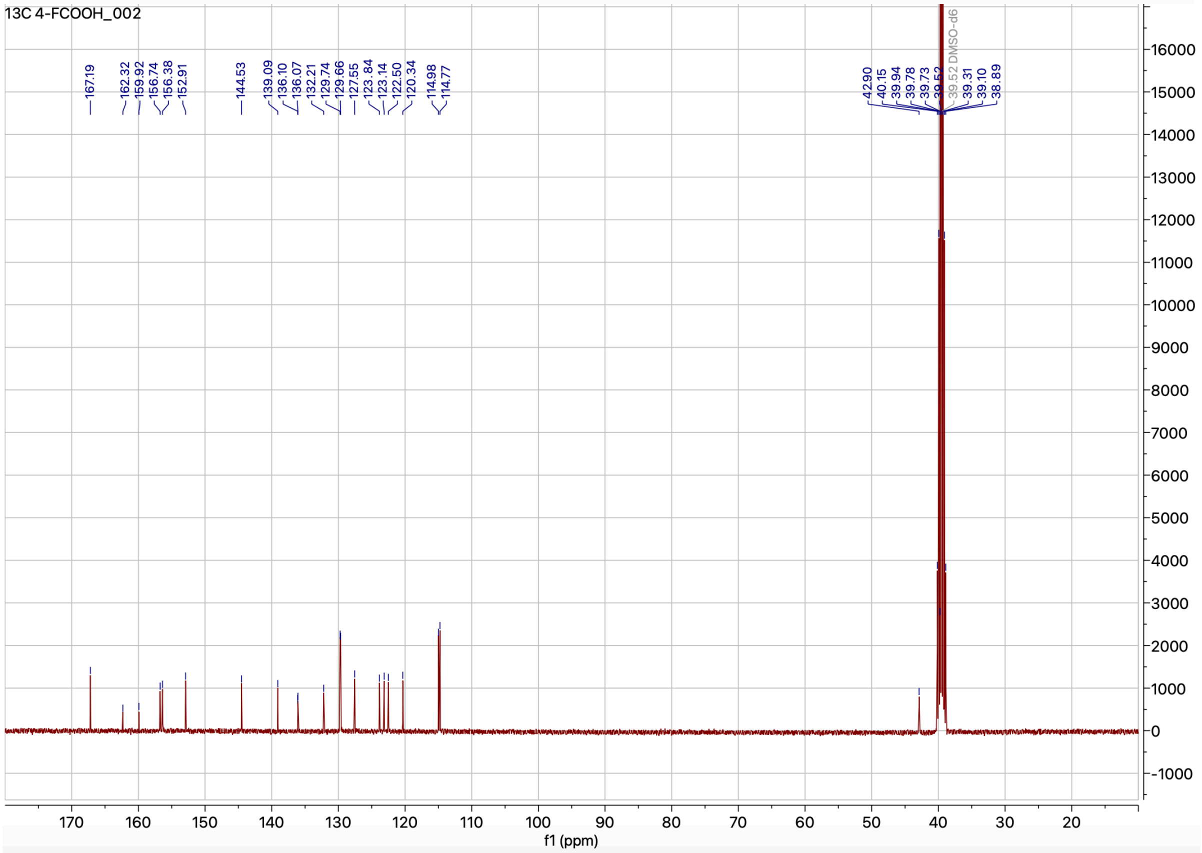

**Figure S9.** $^{1}H$ and $^{13}C$ NMR spectrum of compound **4g**

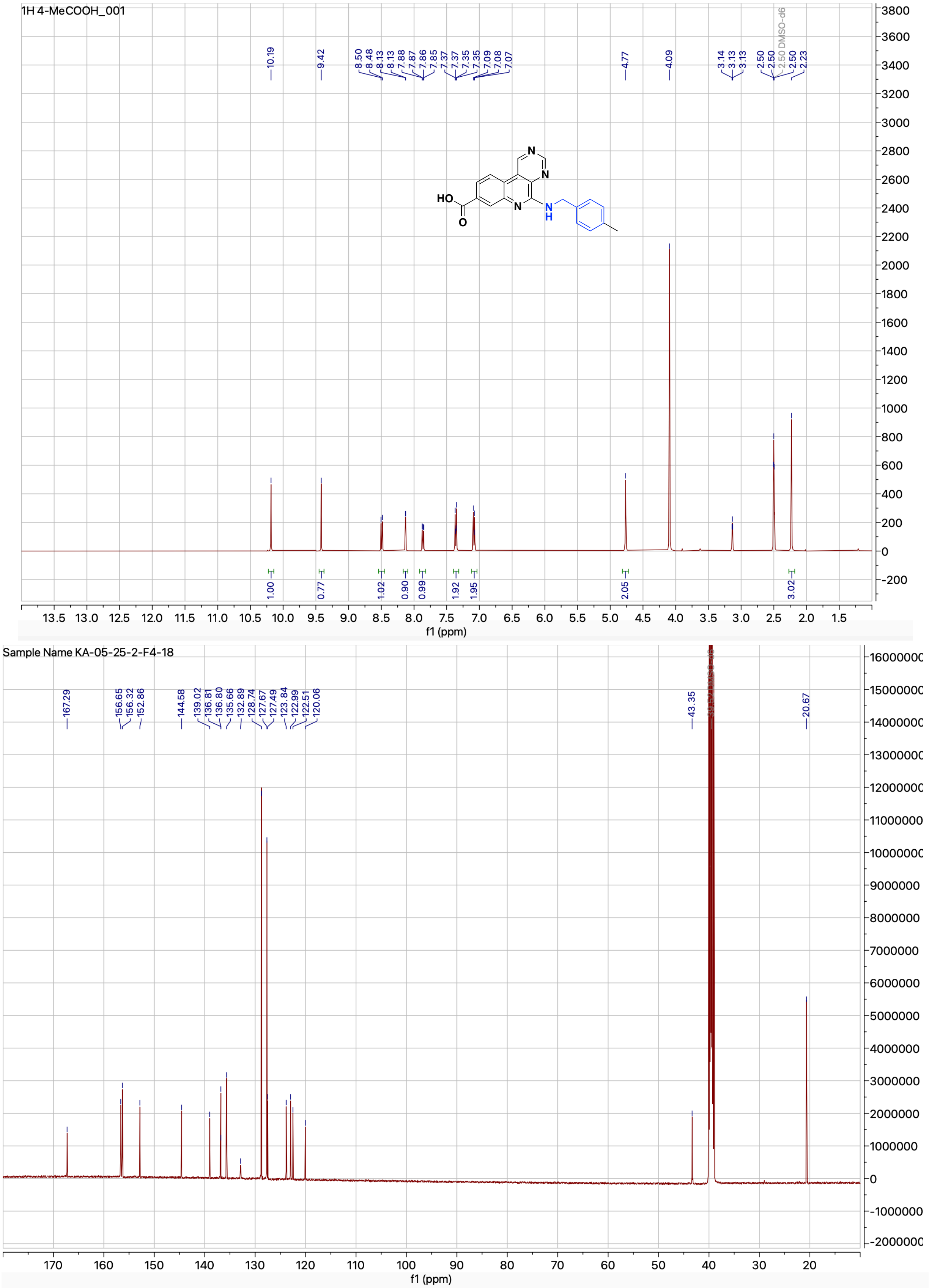


**Figure S10.** $^{1}$H and $^{13}$C NMR spectrum of compound **4h**

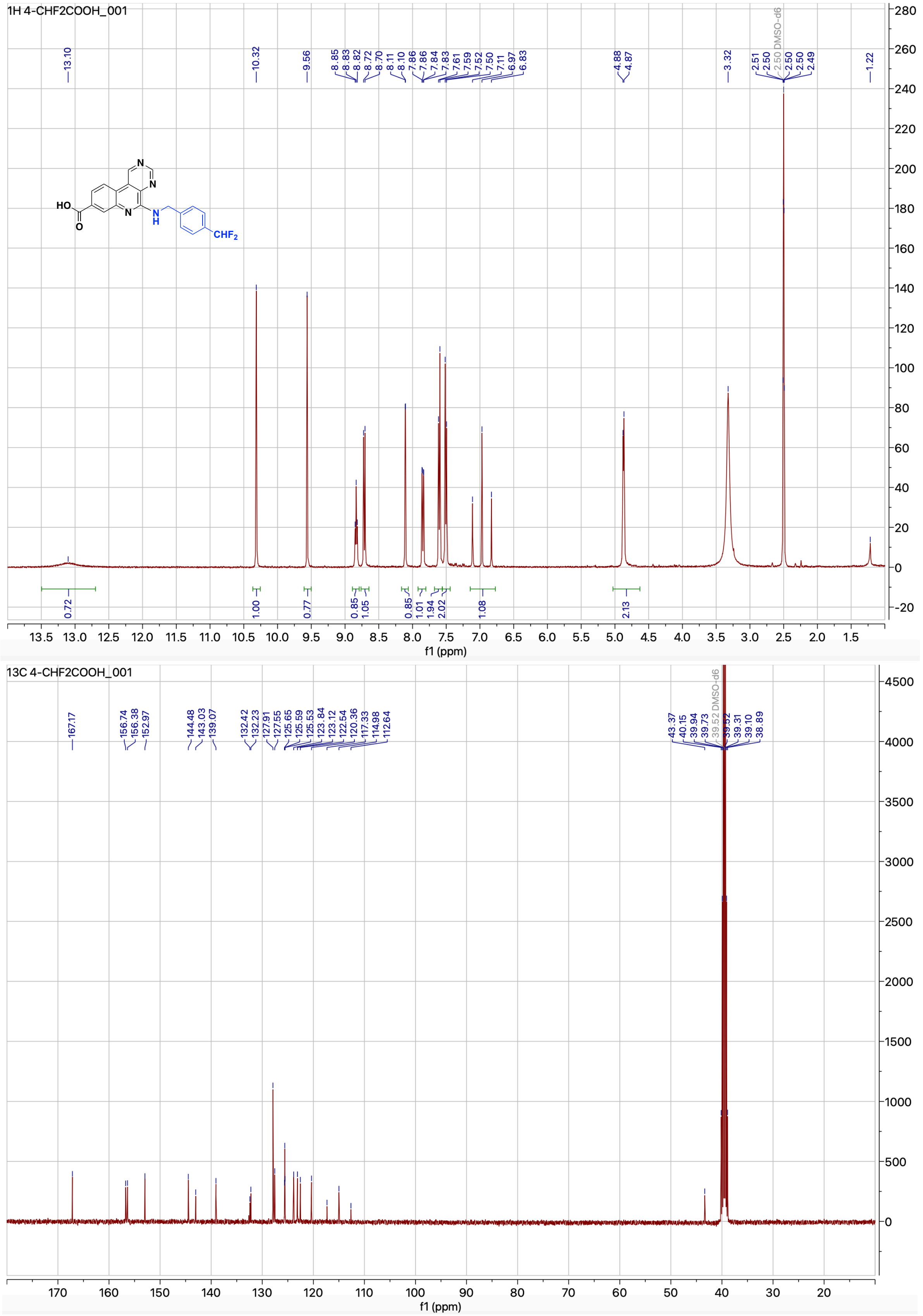


**Figure S11.** $^{1}$H and $^{13}$C NMR spectrum of compound **4i**

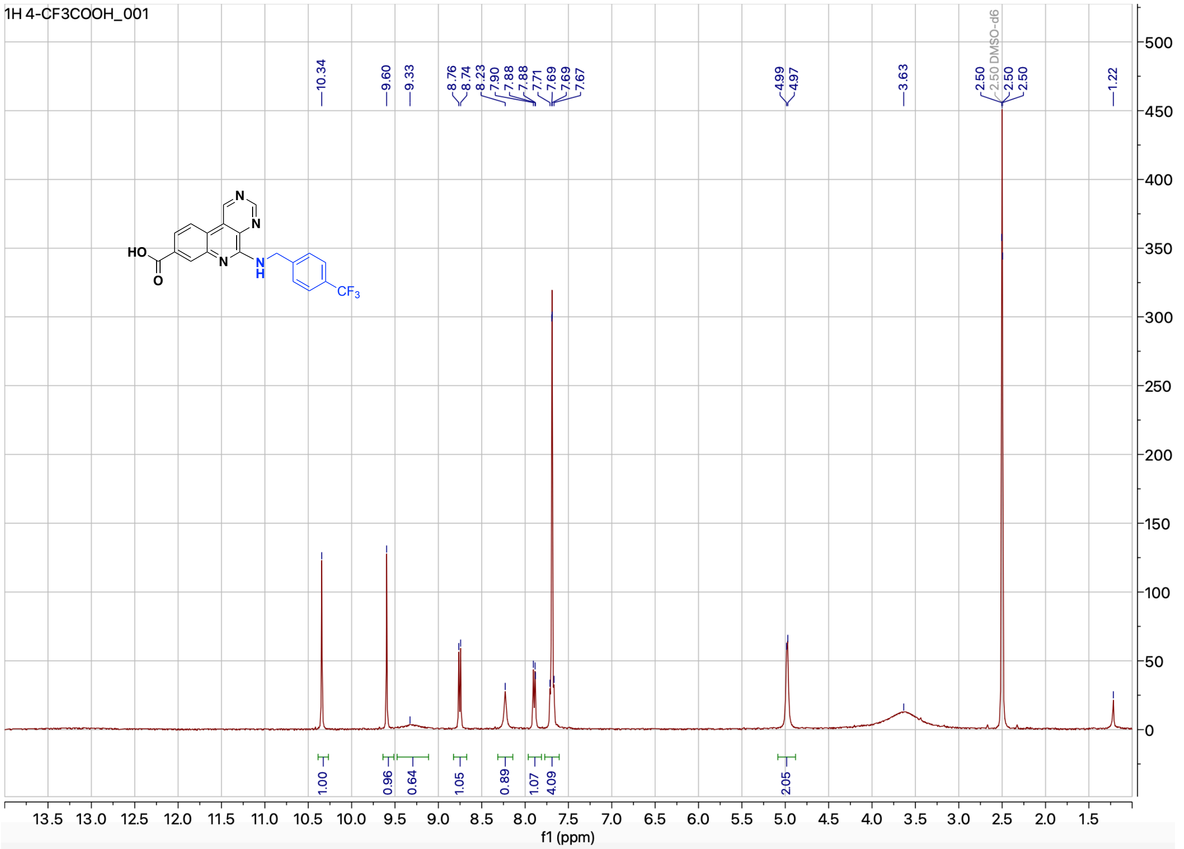


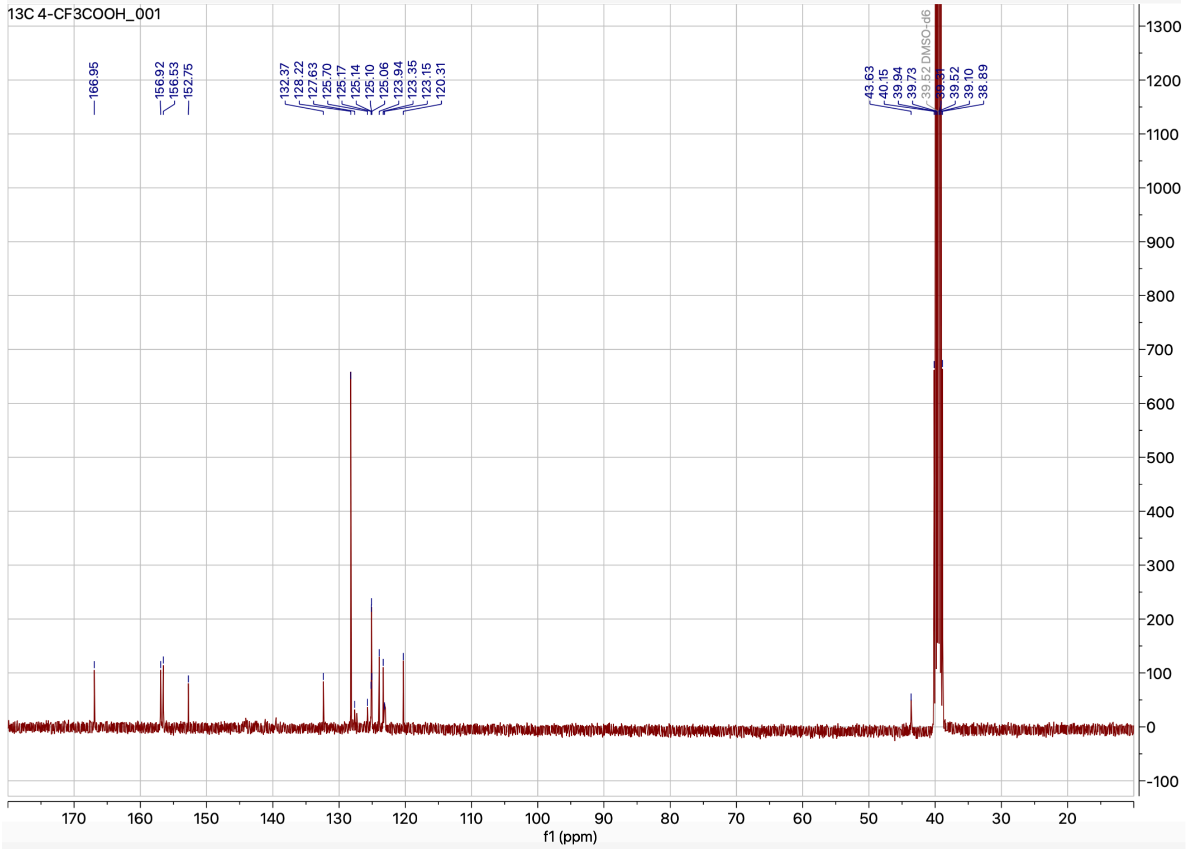


**Figure S12.** $^{1}$H and $^{13}$C NMR spectrum of compound **4j**

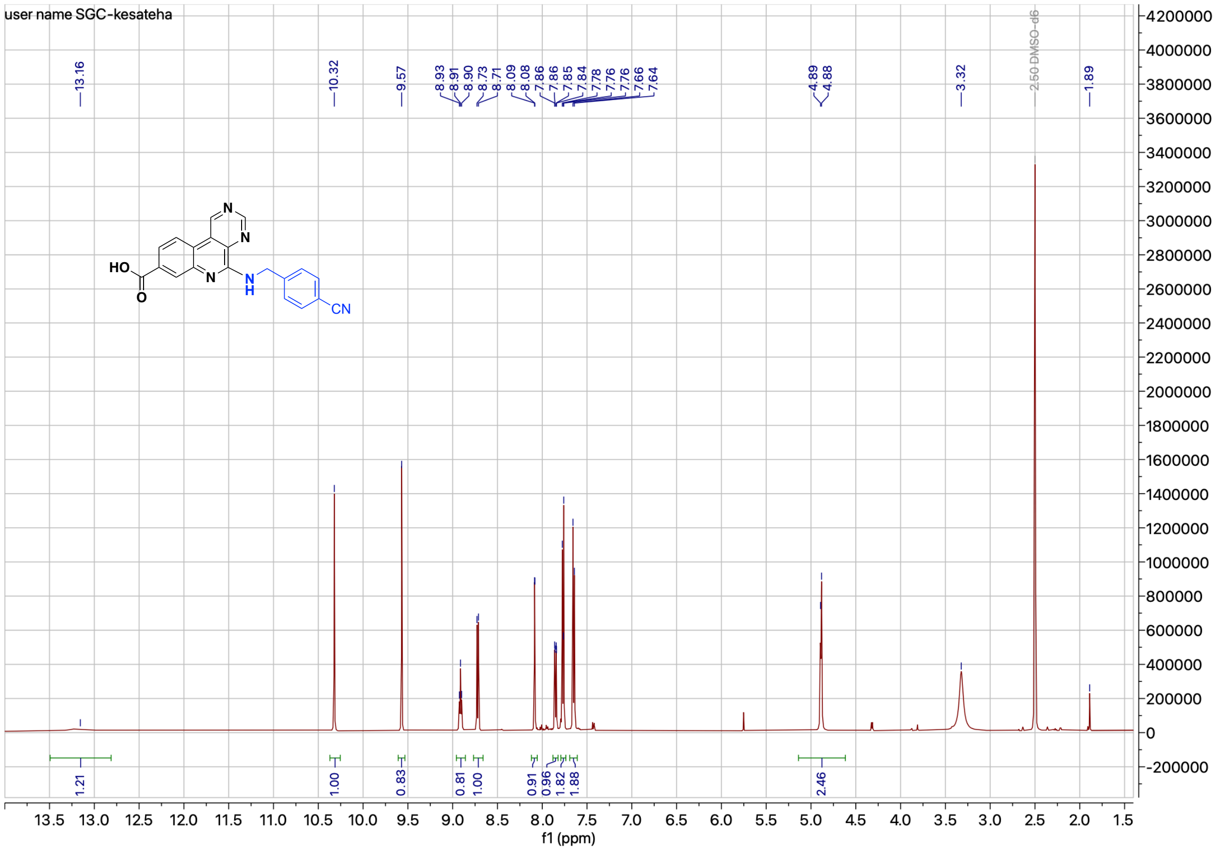


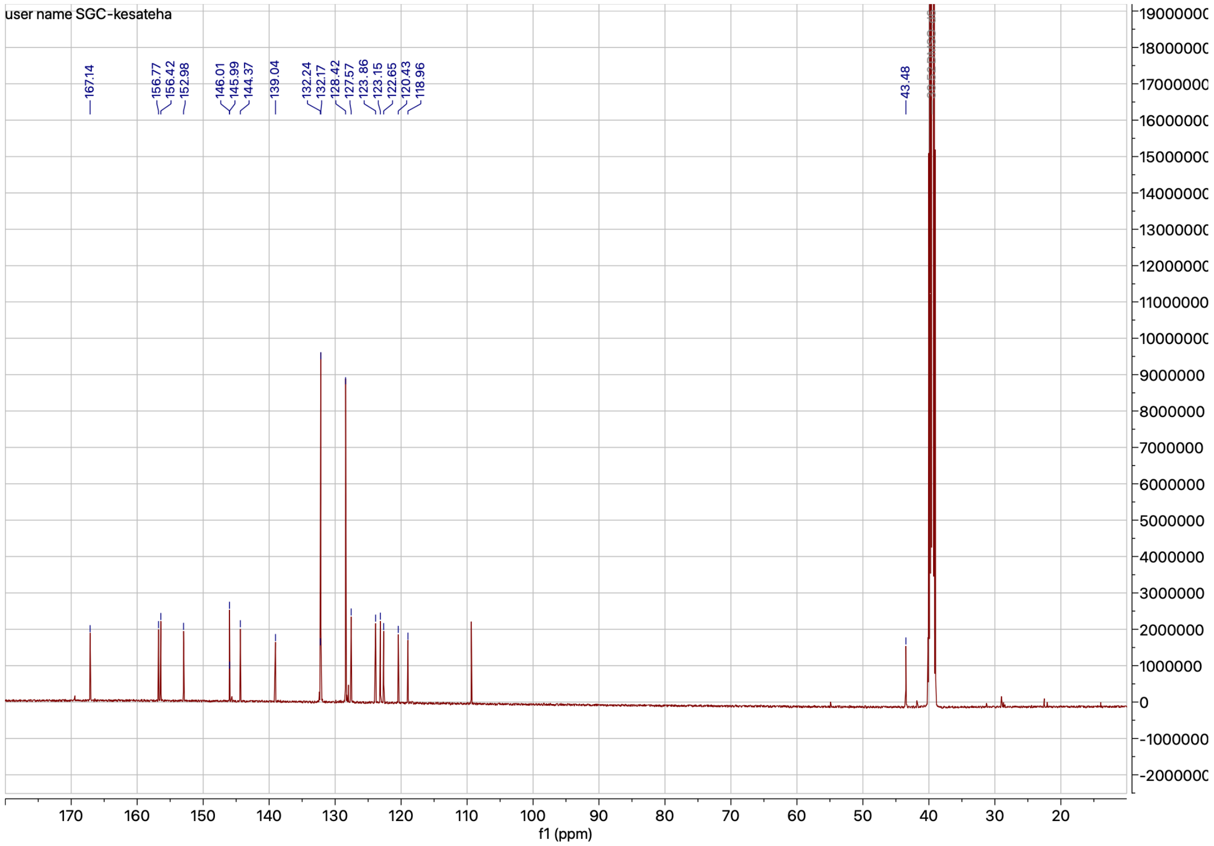


**Figure S13.** $^{1}$H and $^{13}$C NMR spectrum of compound **4k**

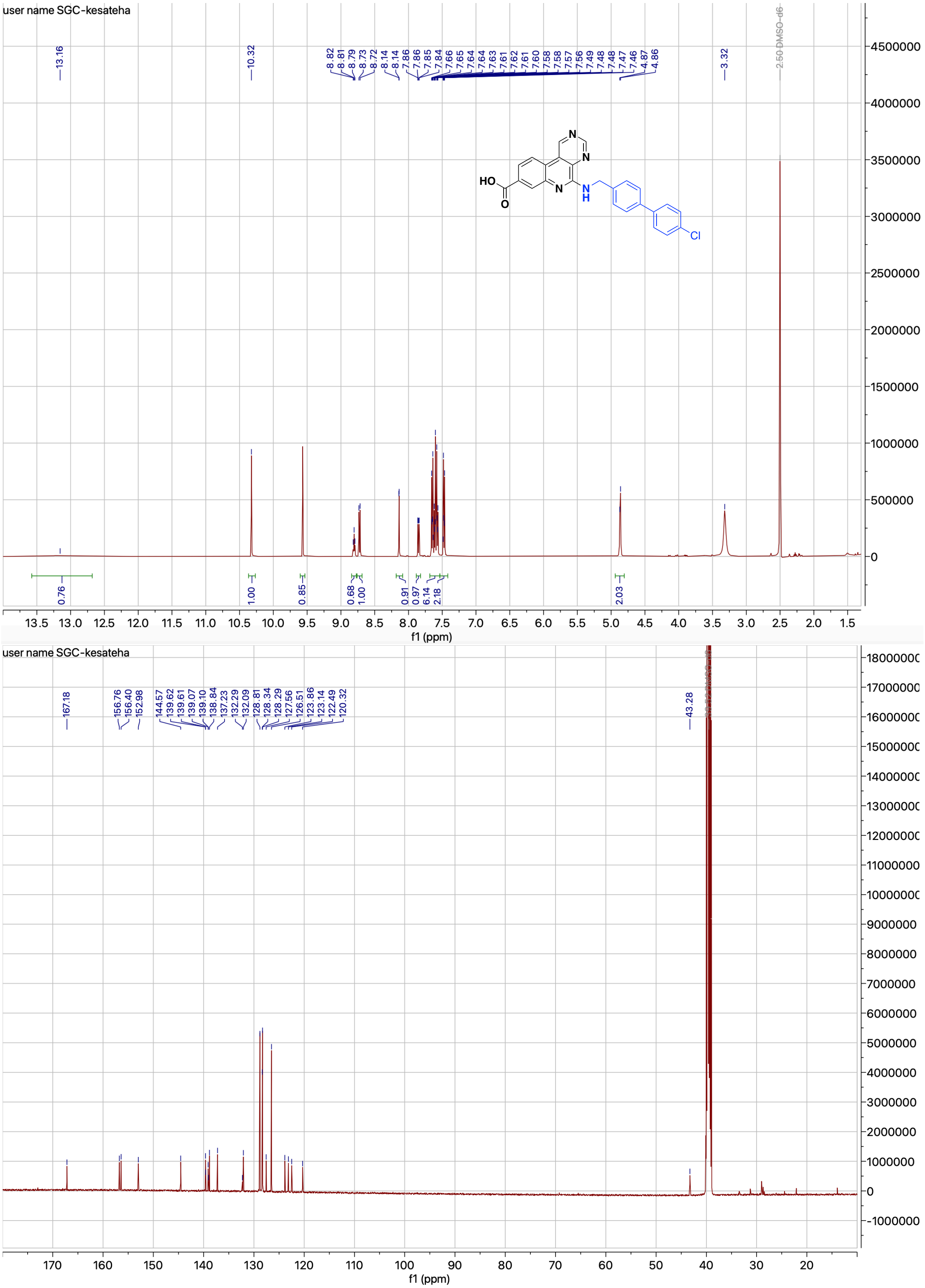


**Figure S14.** $^{1}$H and $^{13}$C NMR spectrum of compound **4l**

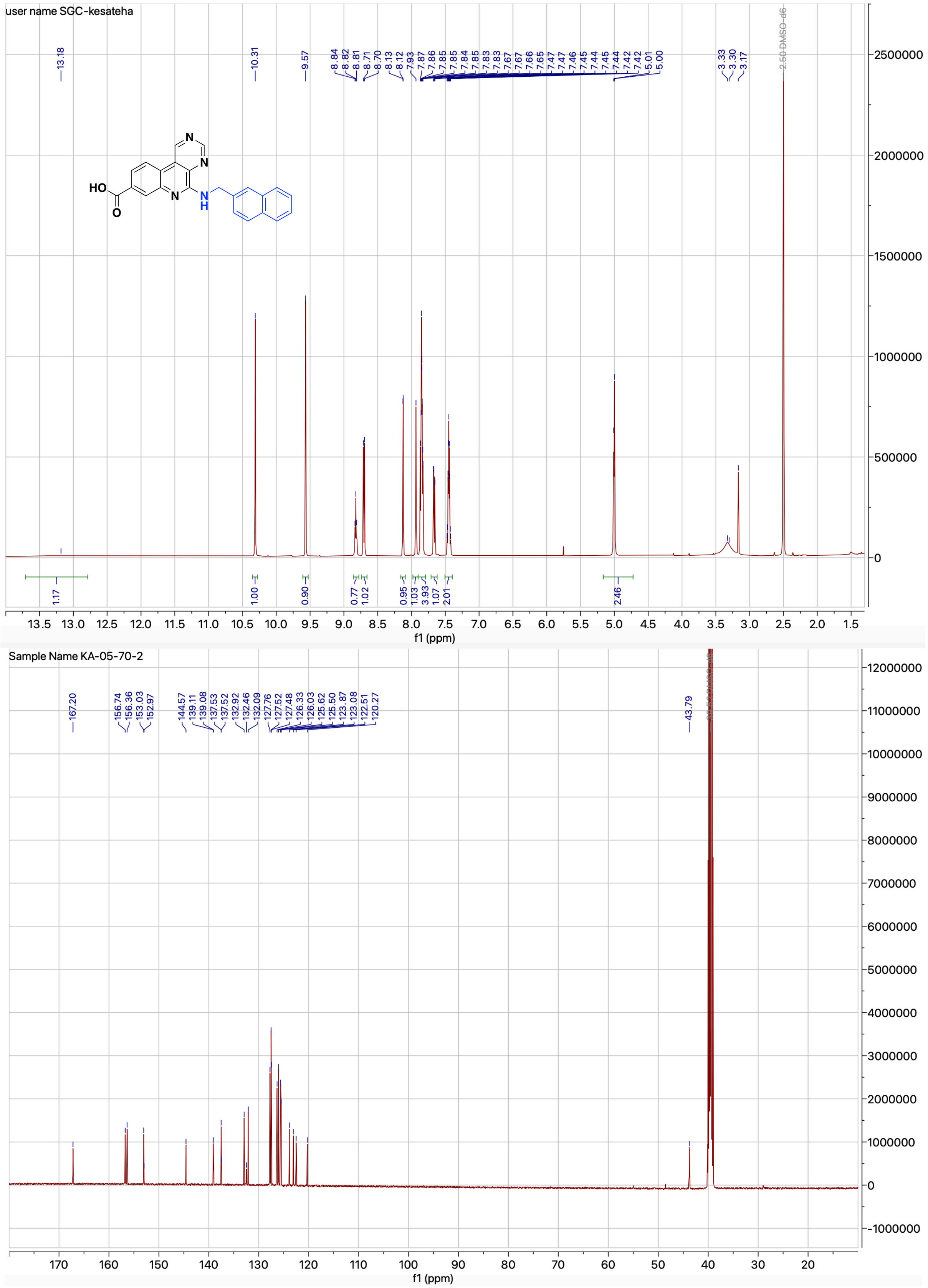


**Figure S15.** $^{1}$H and $^{13}$C NMR spectrum of compound **4m**

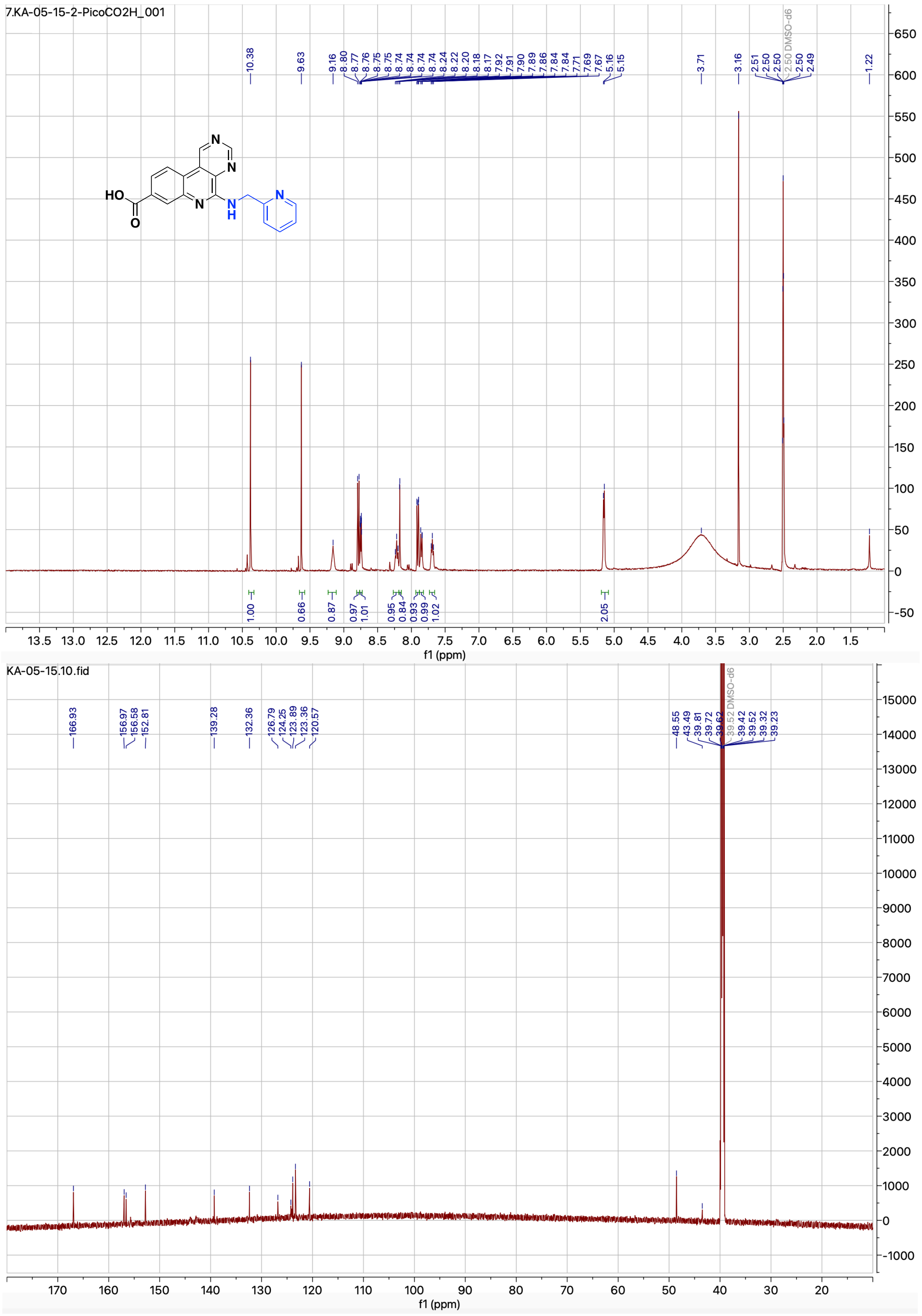


**Figure S16.** $^{1}$H and $^{13}$C NMR spectrum of compound **4n**

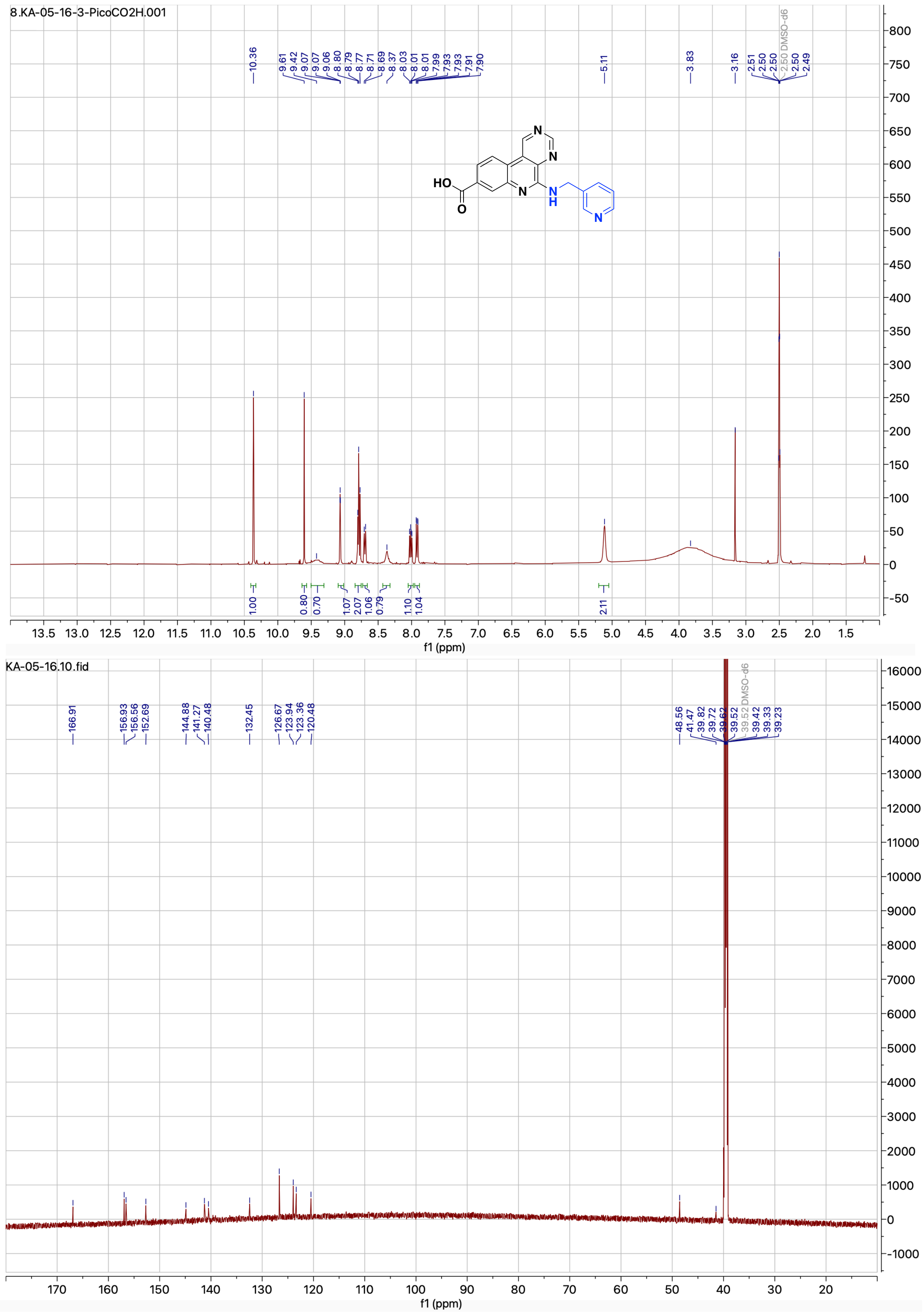


**Figure S17.** $^{1}$H and $^{13}$C NMR spectrum of compound **4o**

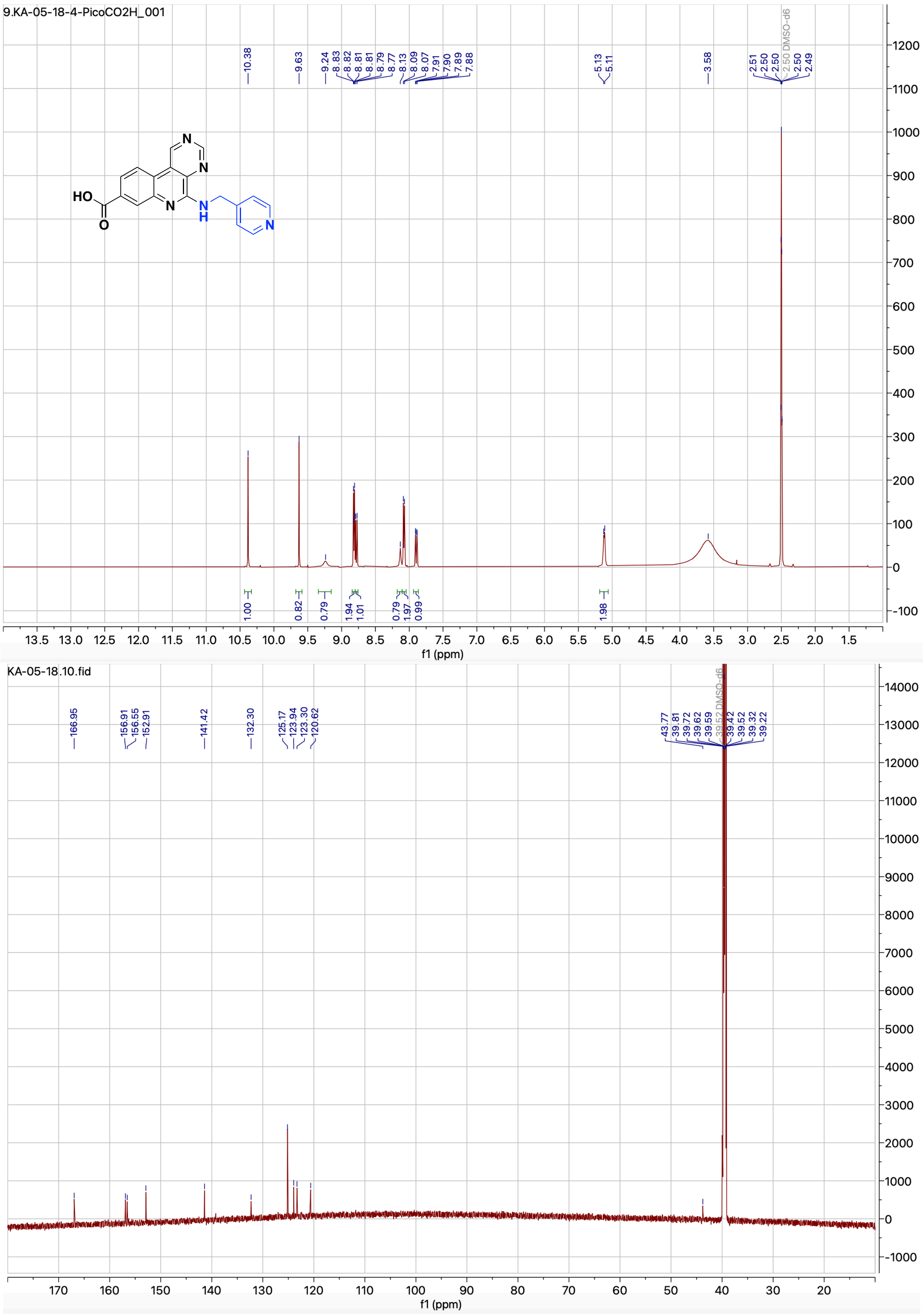


**Figure S18.** $^{1}$H and $^{13}$C NMR spectrum of compound **4p**

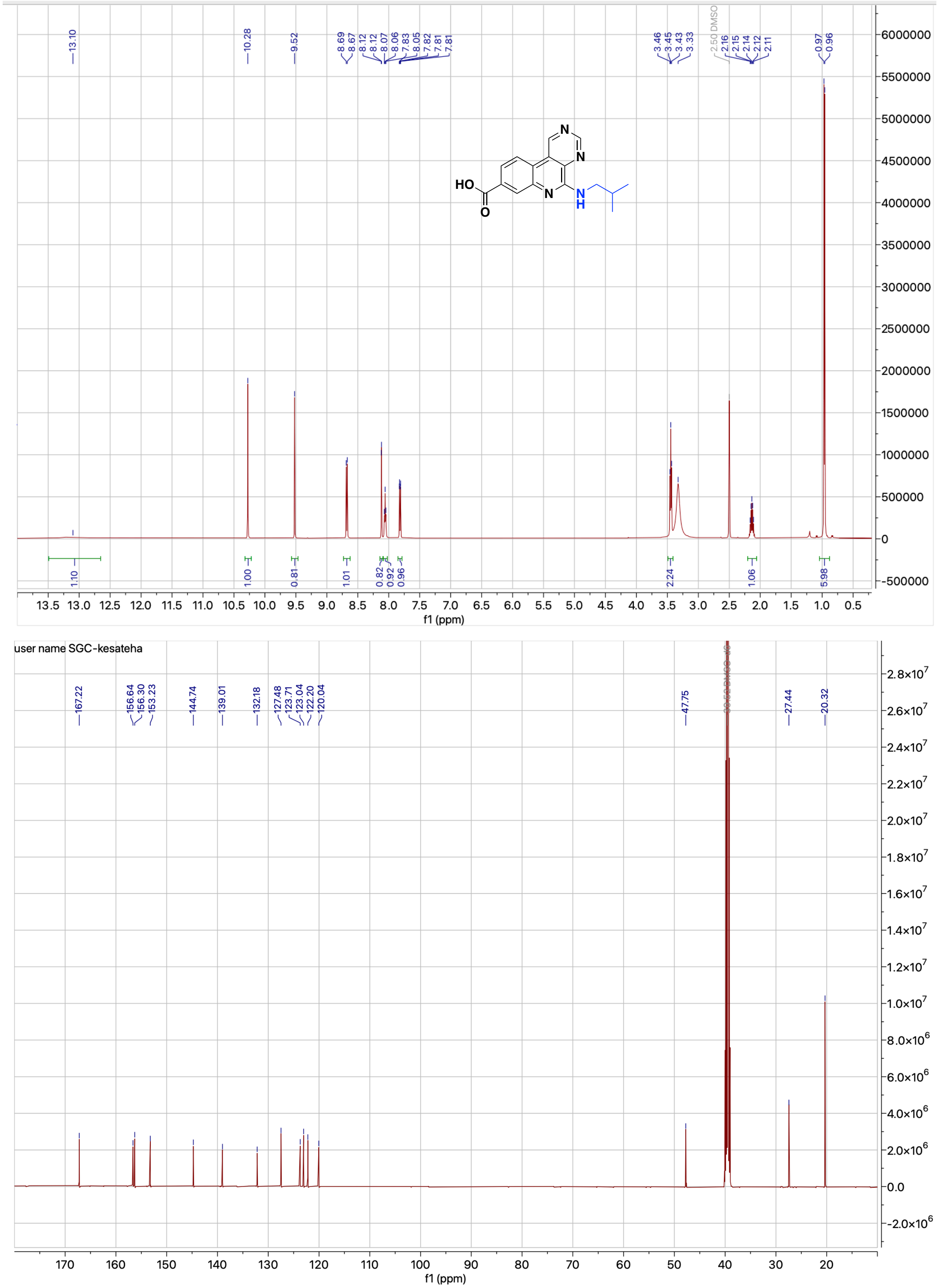


**Figure S19.** $^{1}$H and $^{13}$C NMR spectrum of compound **4q**

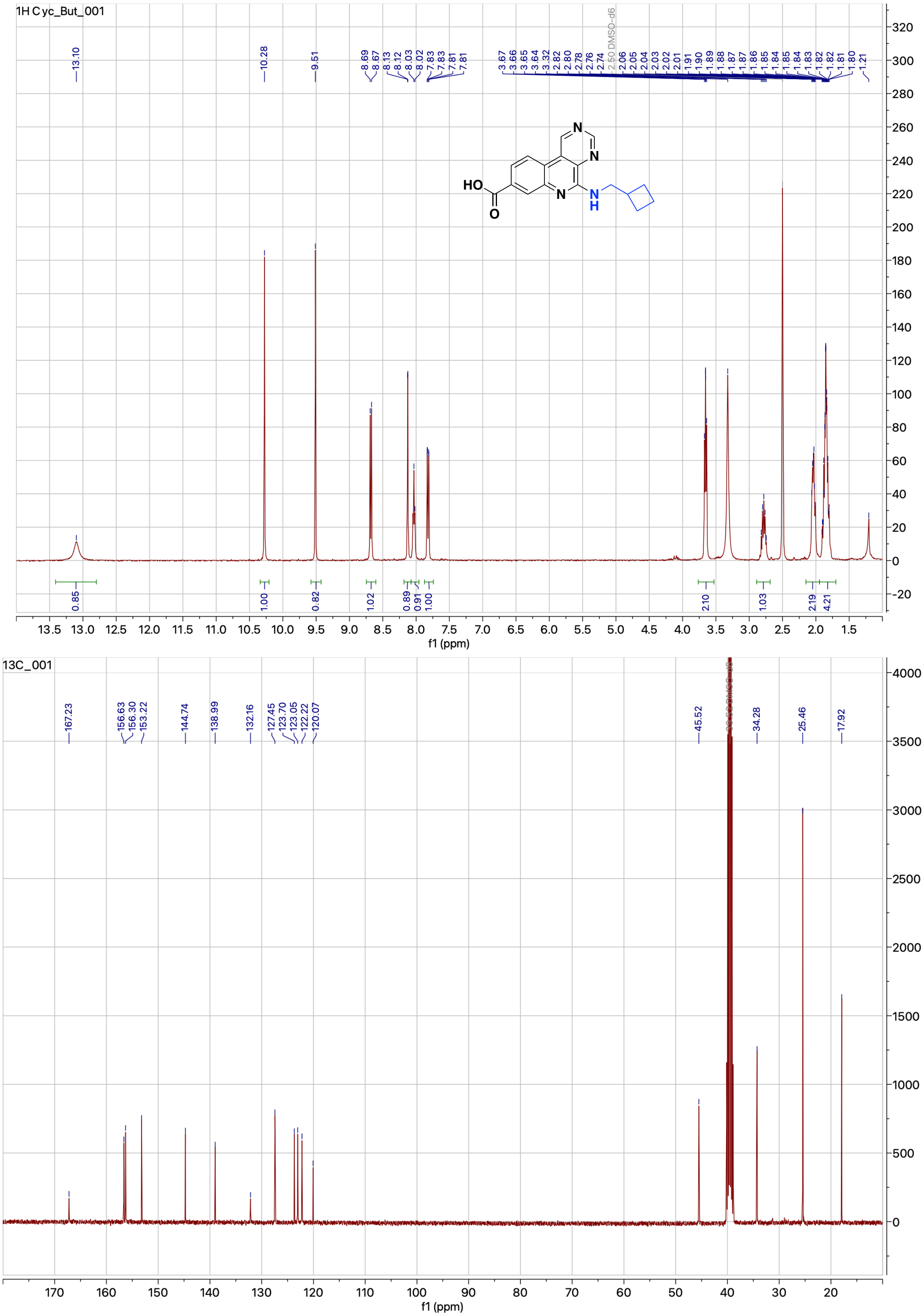


**Figure S20.** $^{1}$H and $^{13}$C NMR spectrum of compound **4r**

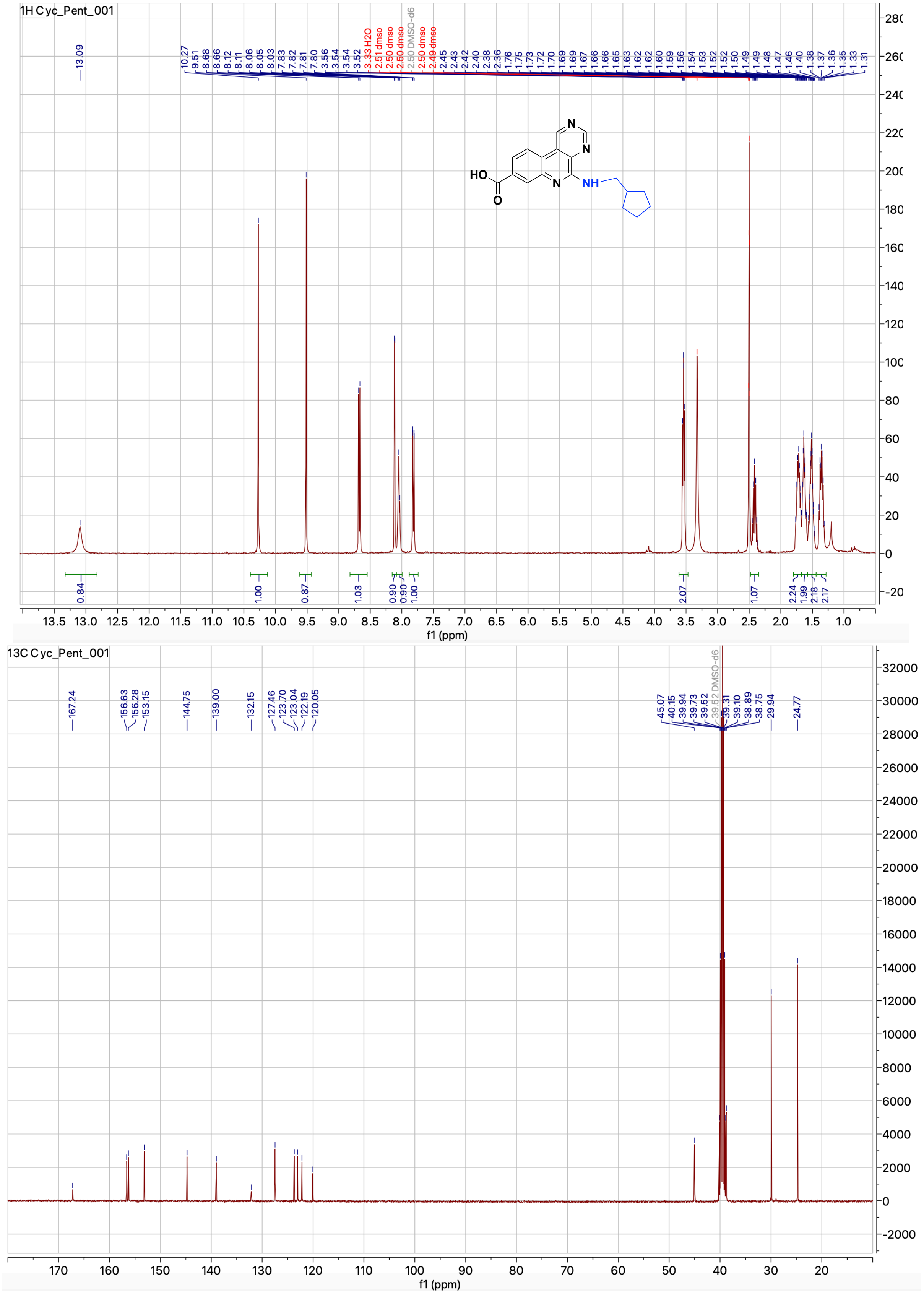


**Figure S21.** $^{1}$H and $^{13}$C NMR spectrum of compound **4s**

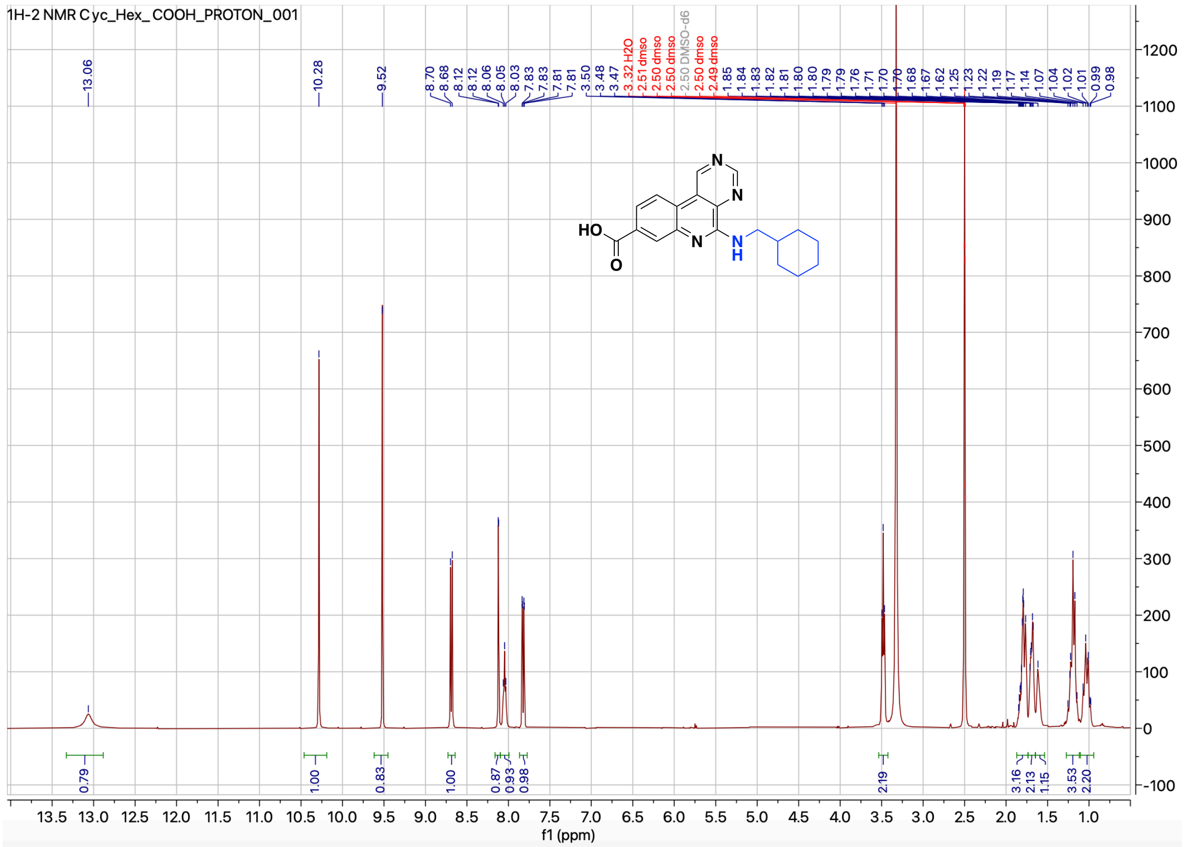


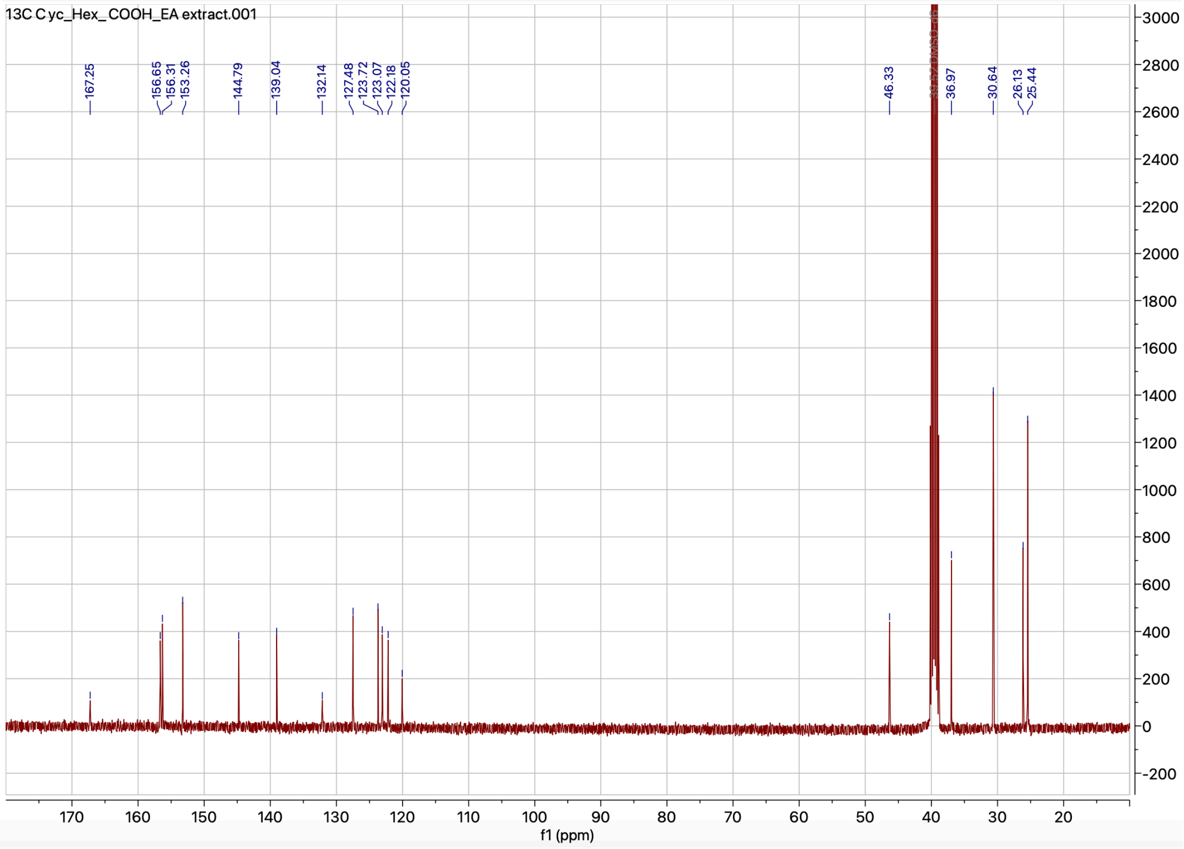


b $^{1}$H and $^{13}$C NMR spectrum of compound **4t**

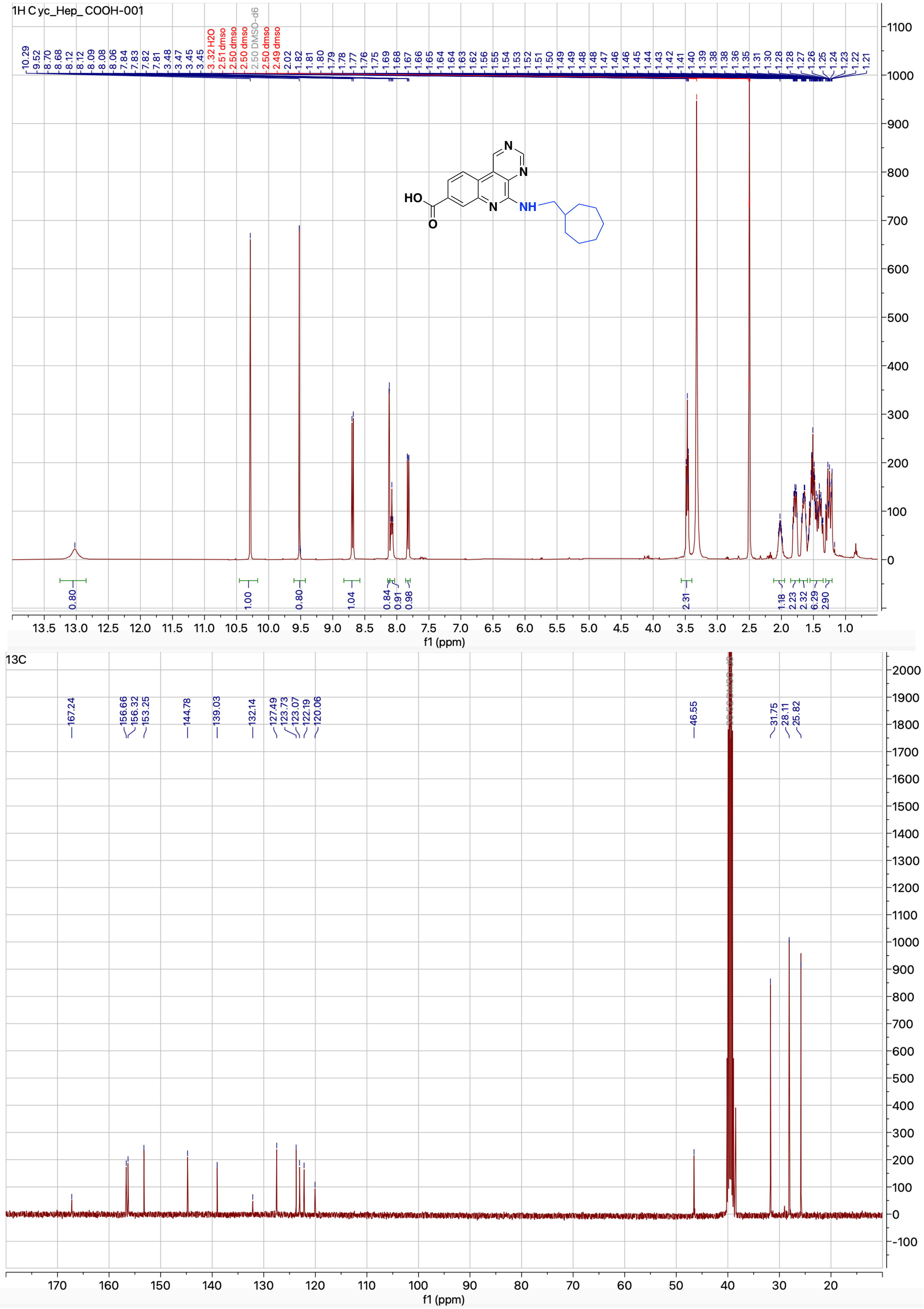

**Figure S23.** $^{1}$H and $^{13}$C NMR spectrum of compound **4u**

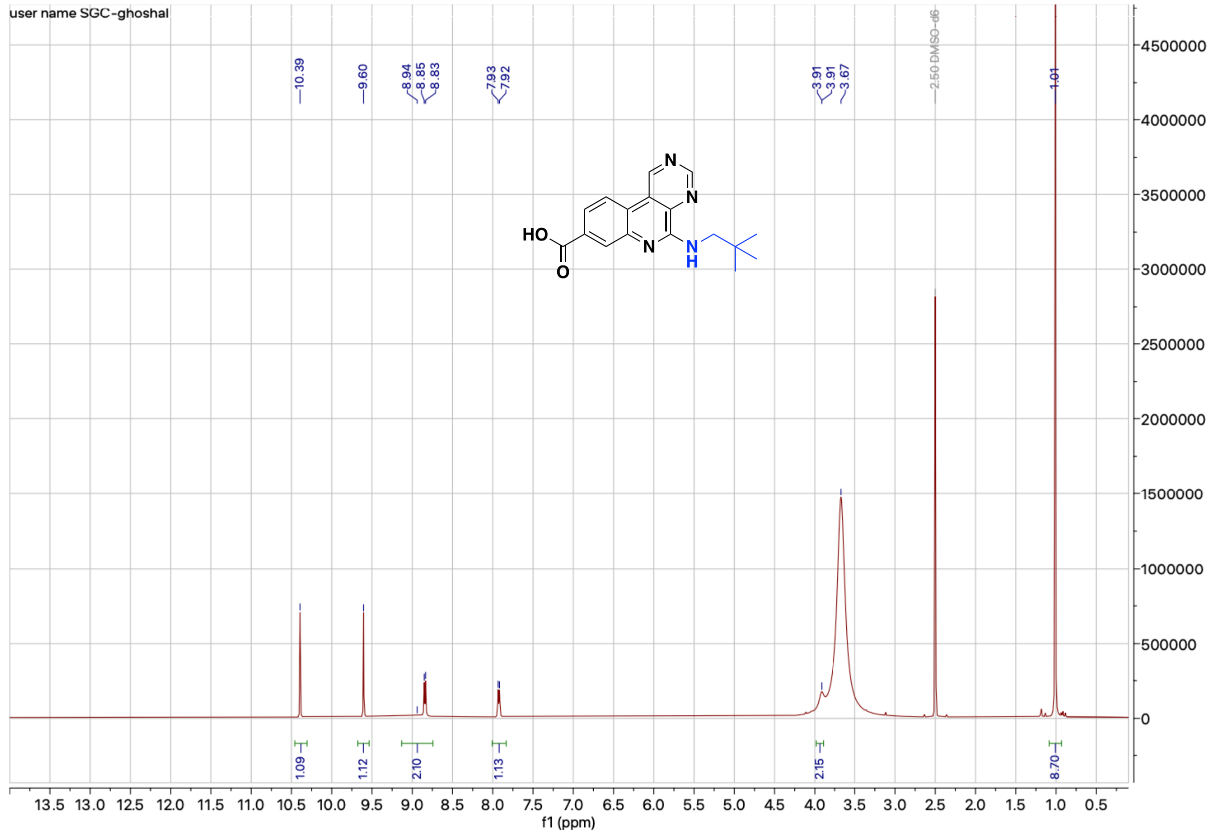


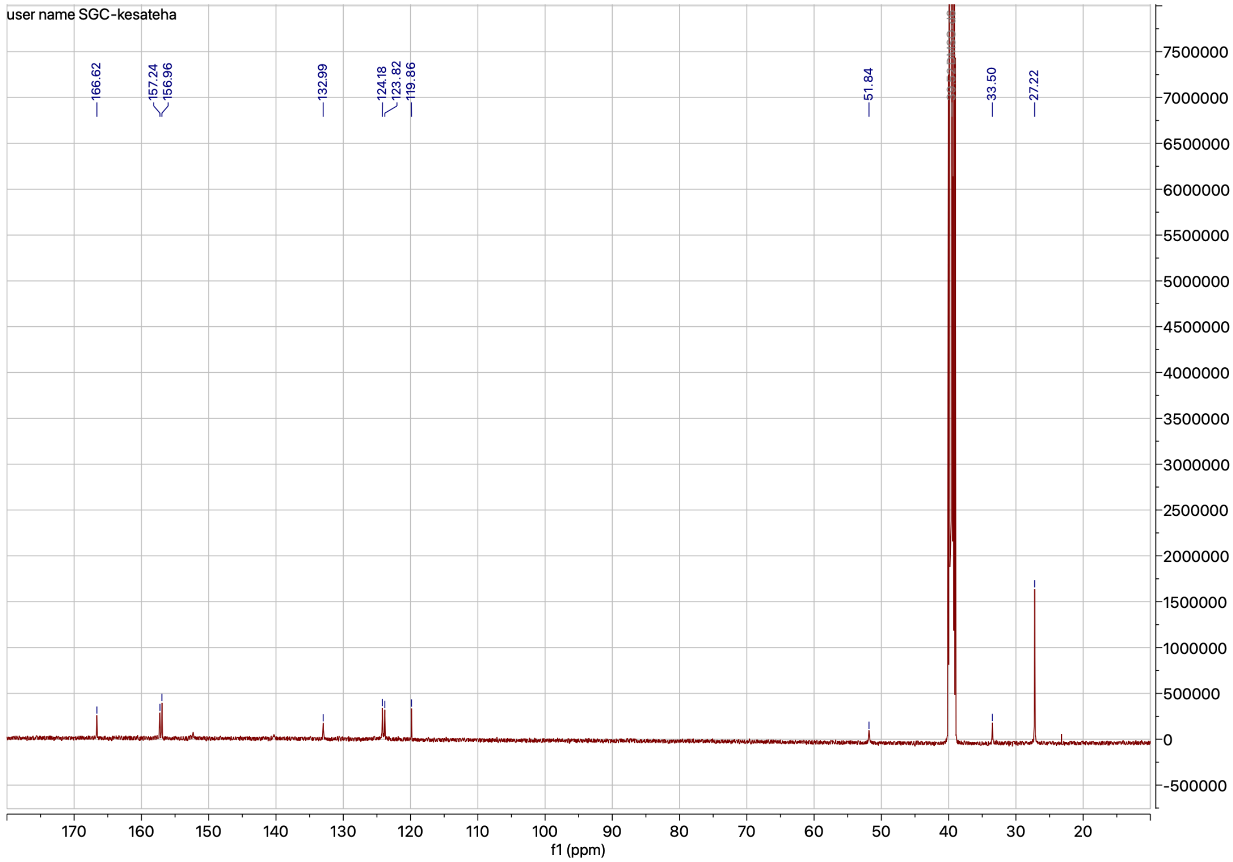


**Figure S24.** $^{1}$H and $^{13}$C NMR spectrum of compound **4v**

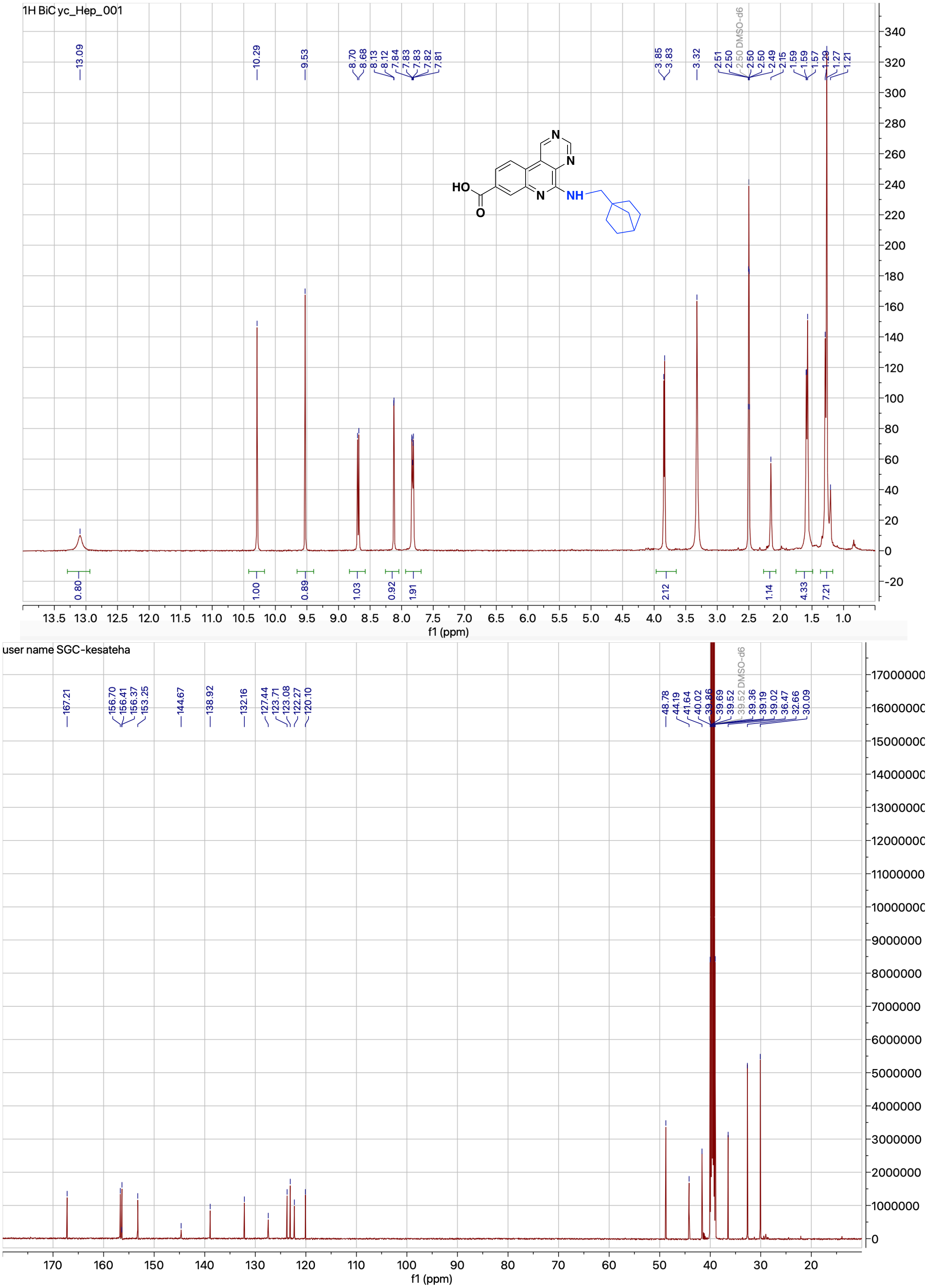


**Figure S25.** $^{1}$H and $^{13}$C NMR spectrum of compound **4w**

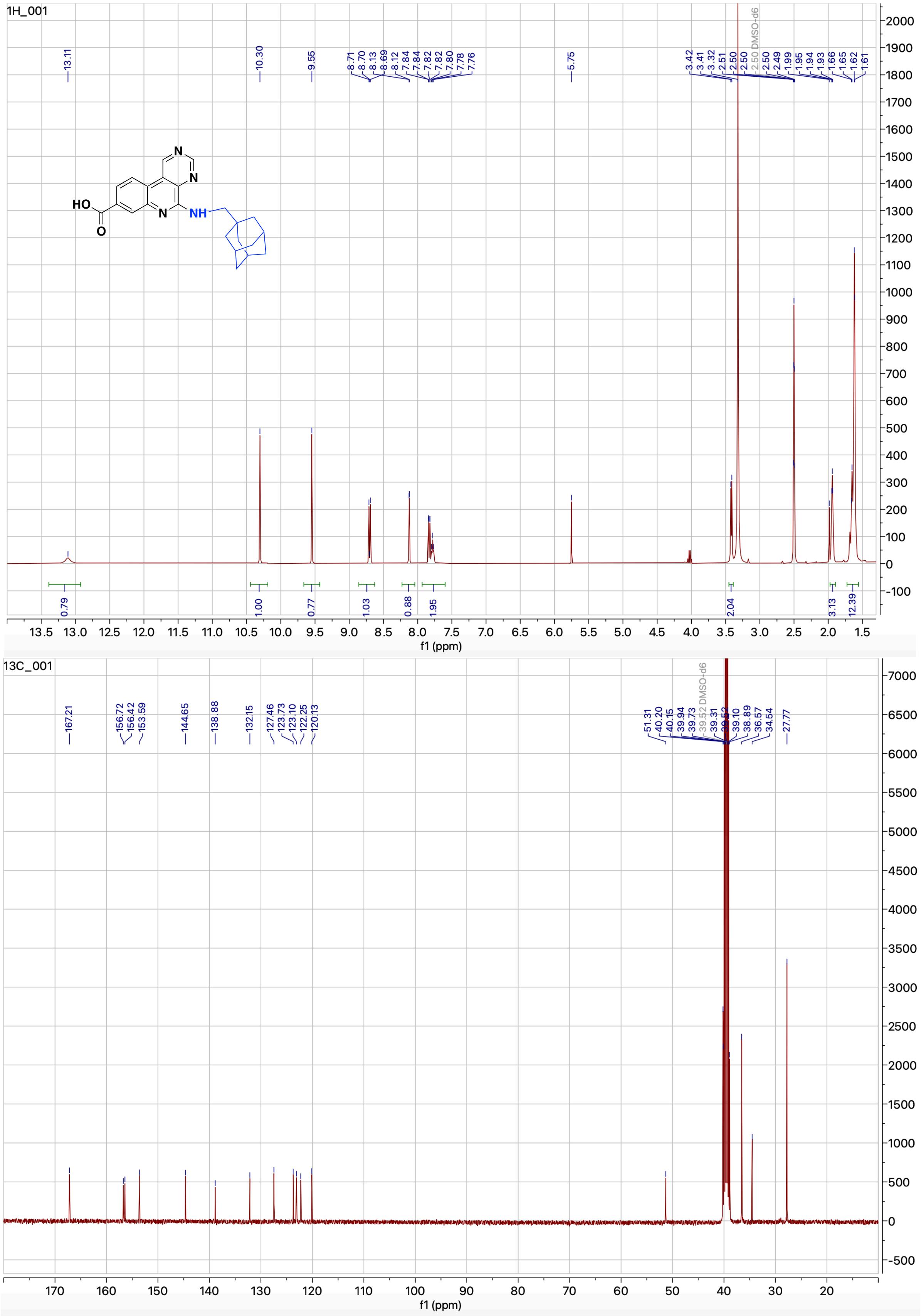


**Figure S26.** $^{1}$H and $^{13}$C NMR spectrum of compound **4x**

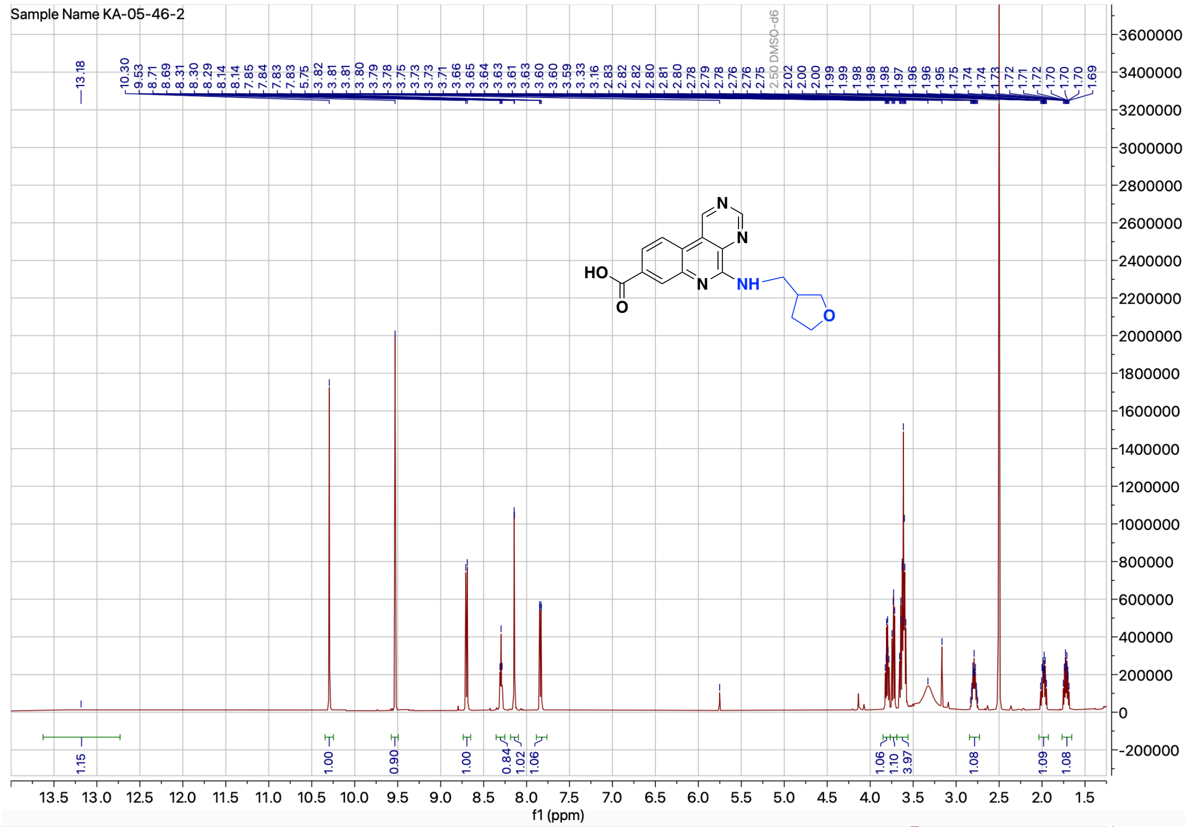


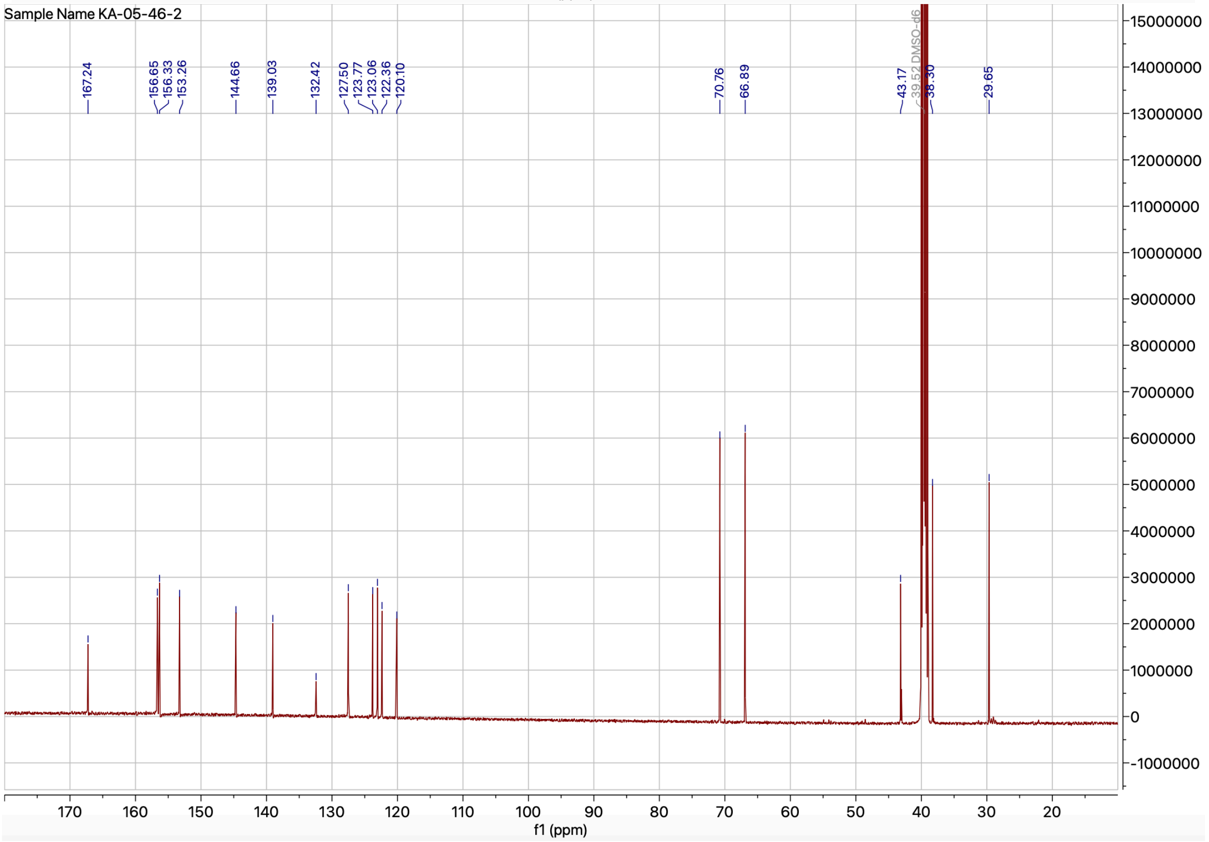


**Figure S27.** $^{1}$H and $^{13}$C NMR spectrum of compound **4y**

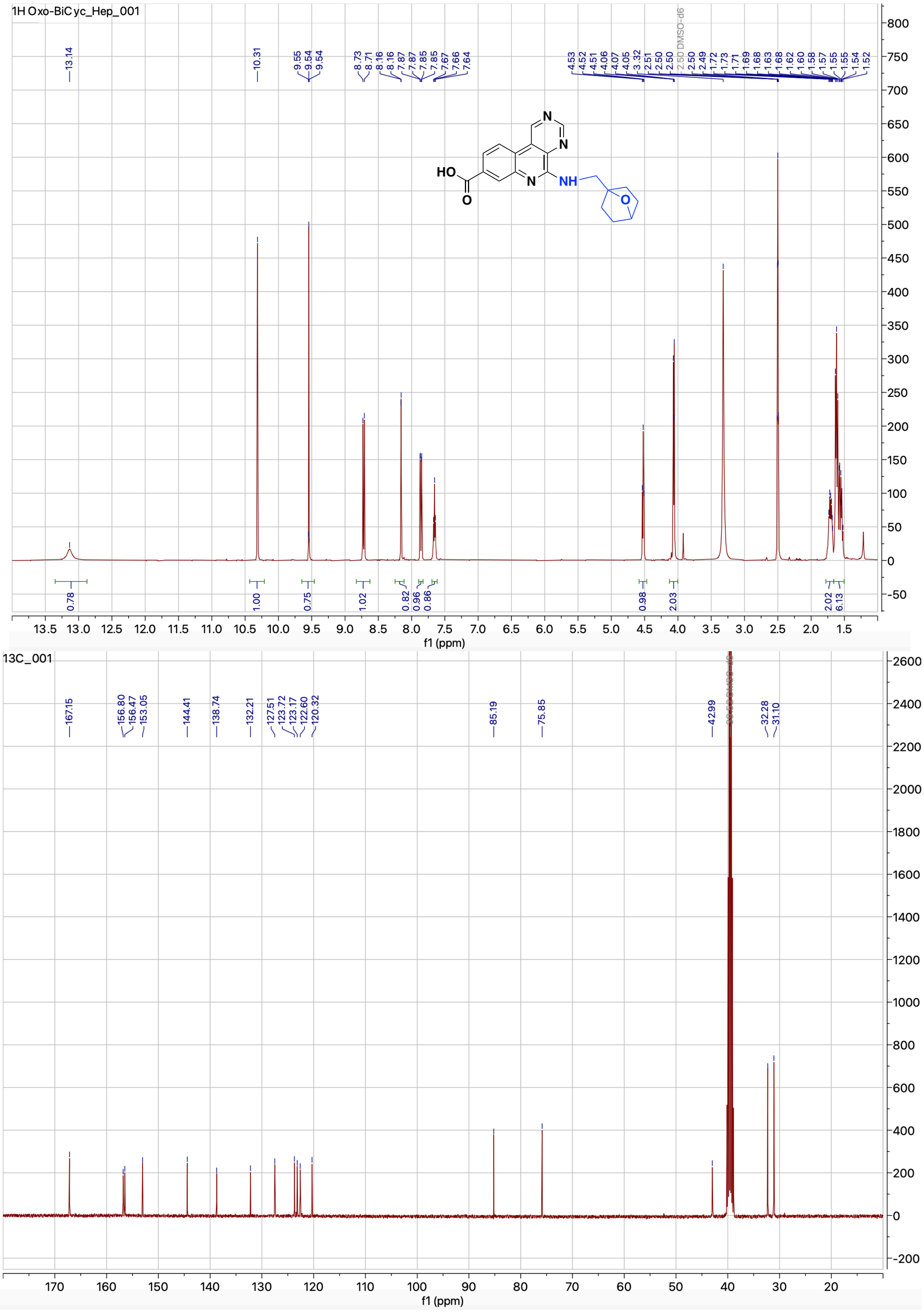


**Figure S28.** $^1$H and $^{13}$C NMR spectrum of compound **4z**

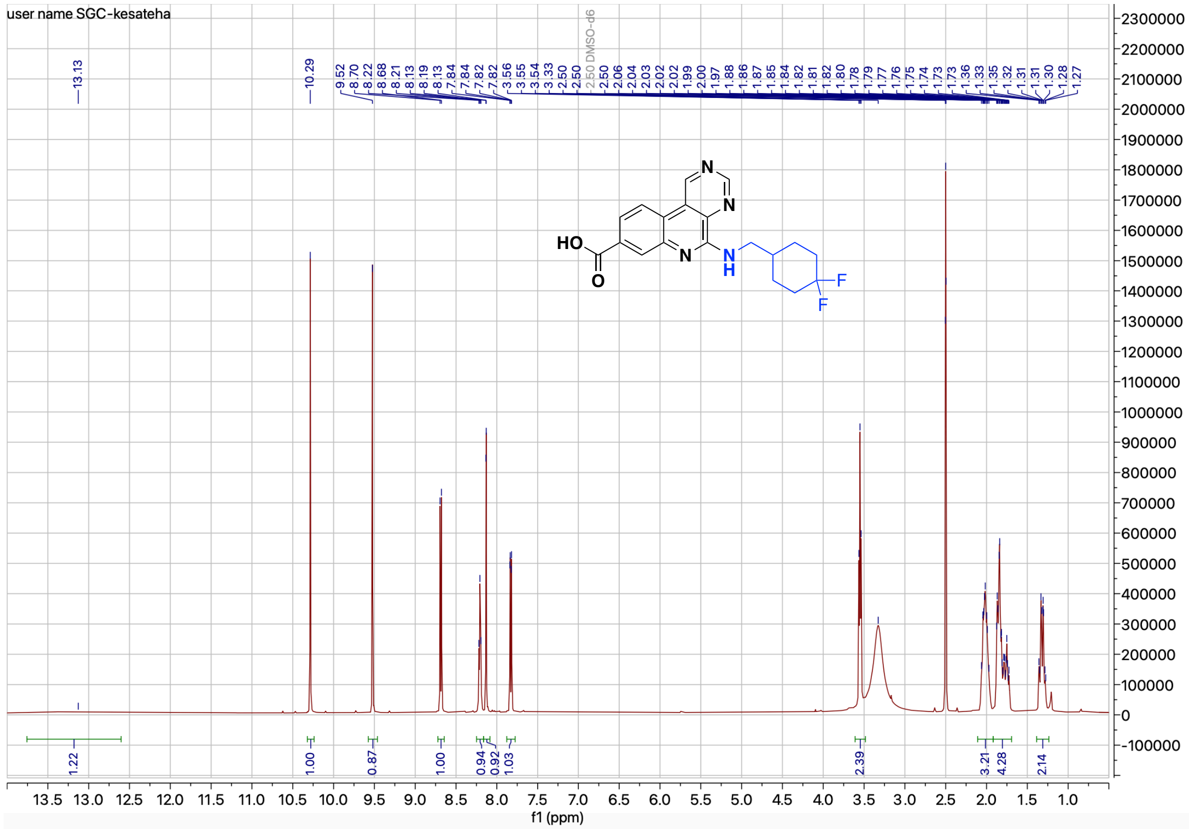


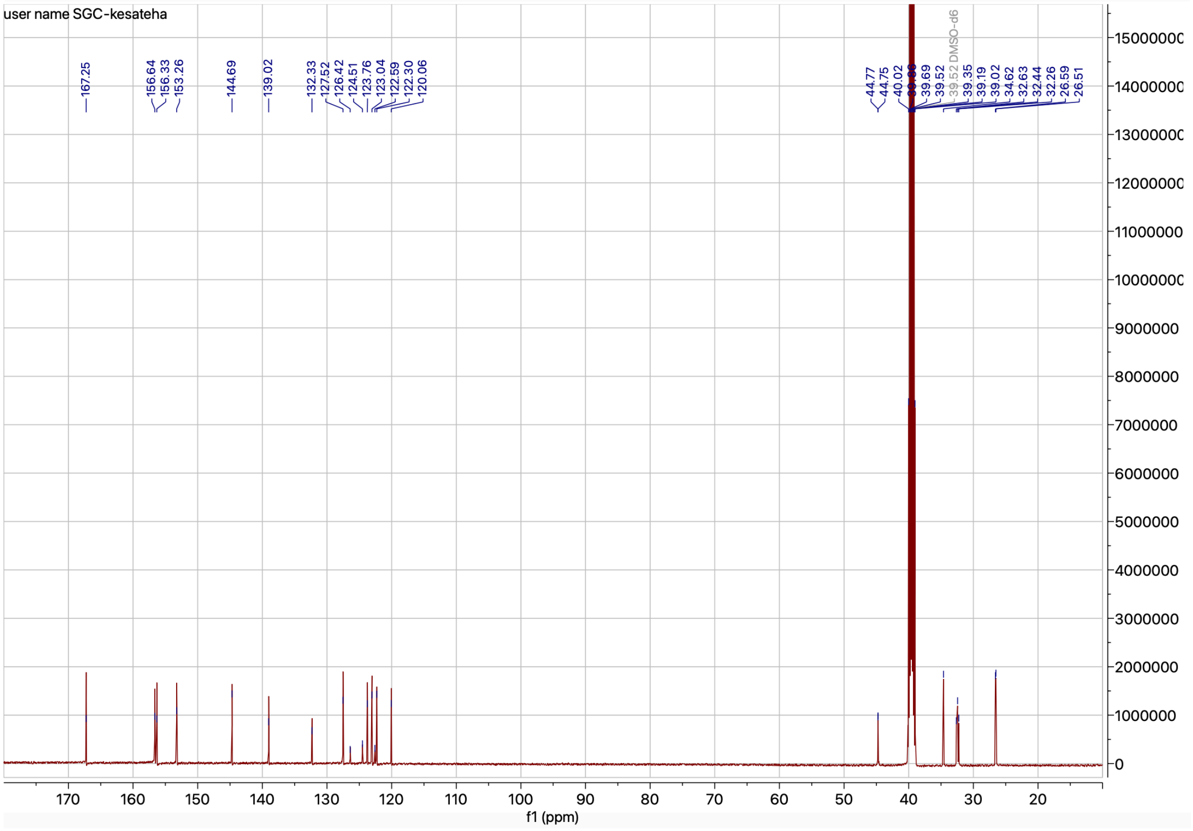


**Figure S29.** $^{1}$H and $^{13}$C NMR spectrum of compound **4aa**

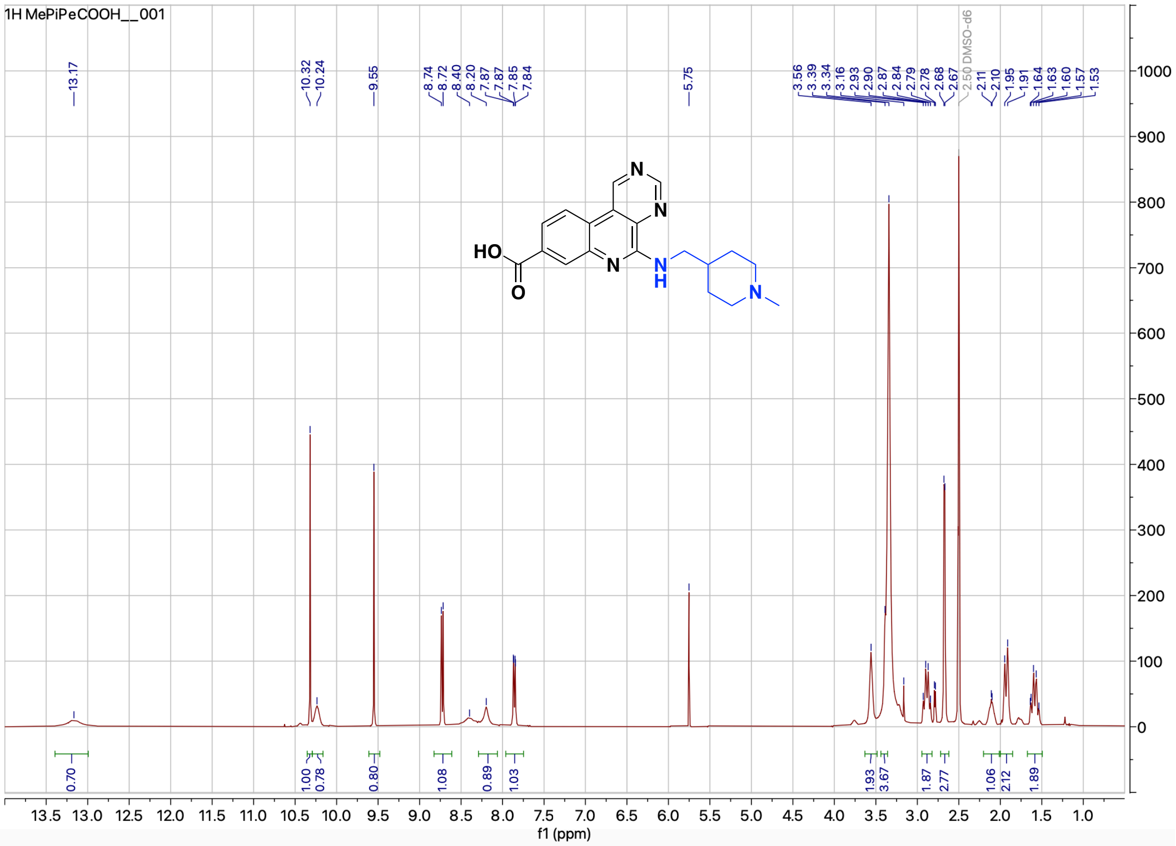


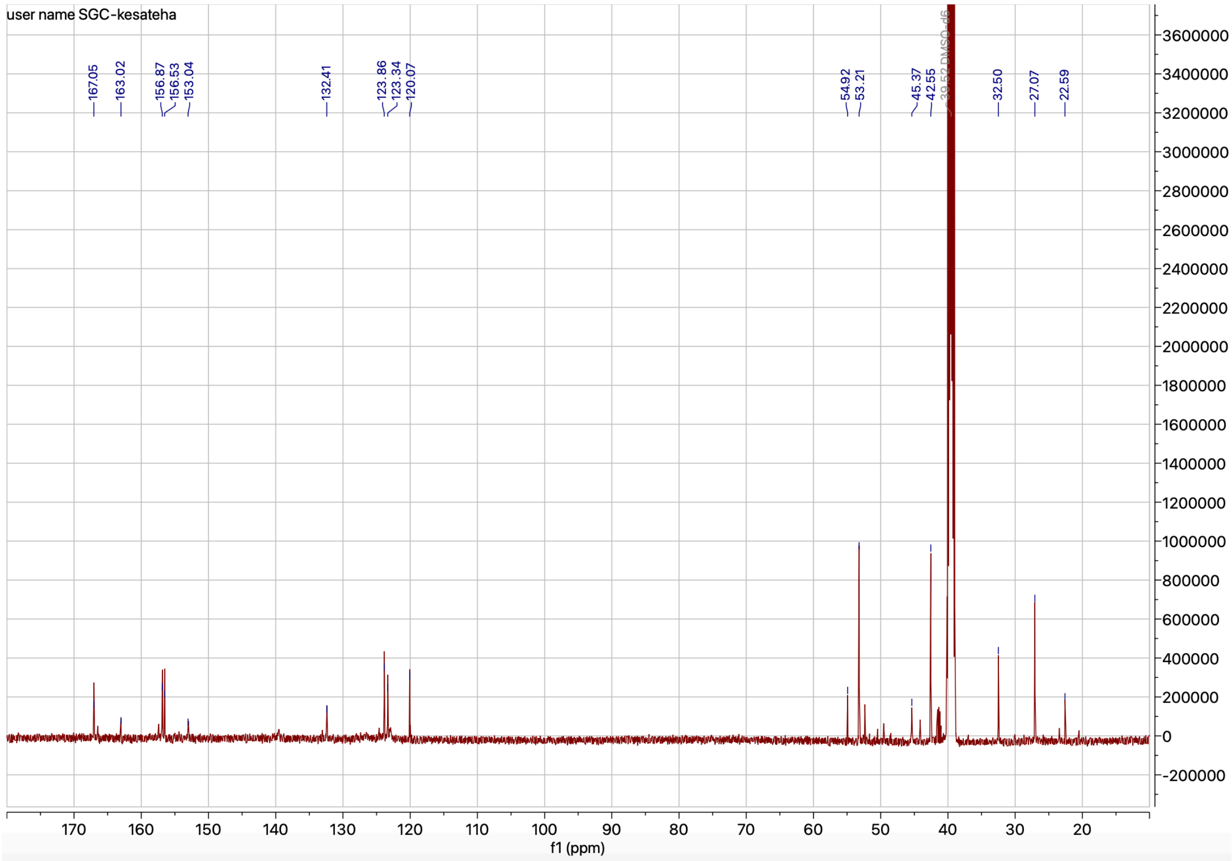


**Figure S30.** $^{1}$H and $^{13}$C NMR spectrum of compound **4ab**

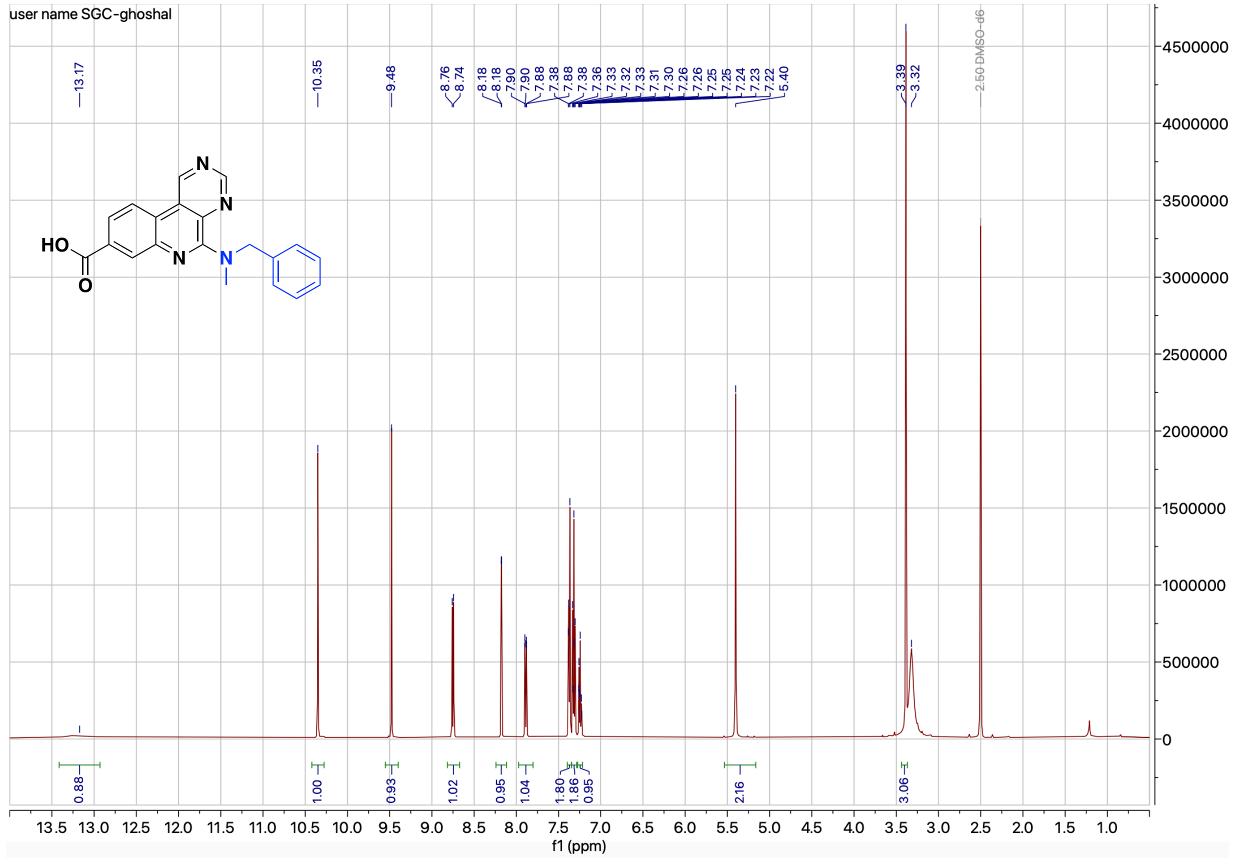


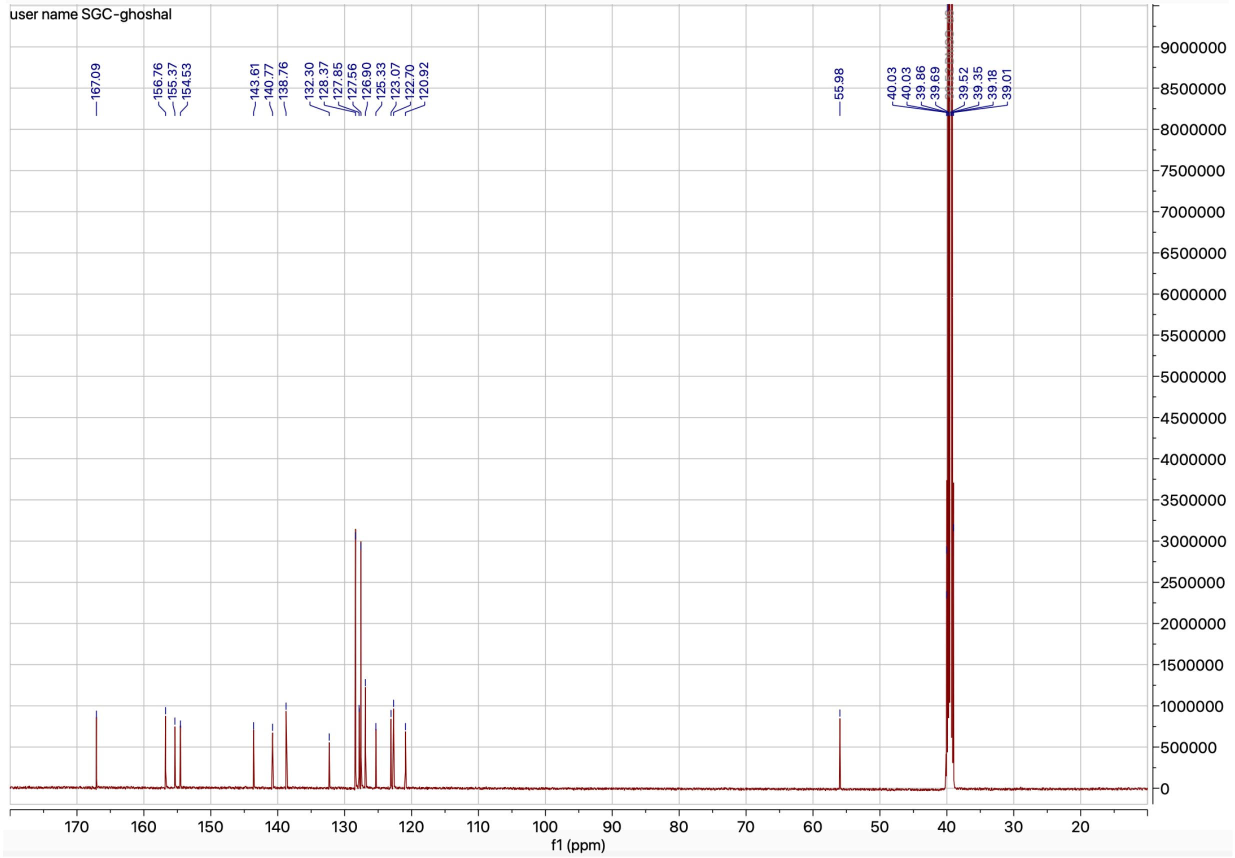


**Figure S31.** $^{1}$H and $^{13}$C NMR spectrum of compound **4ac**

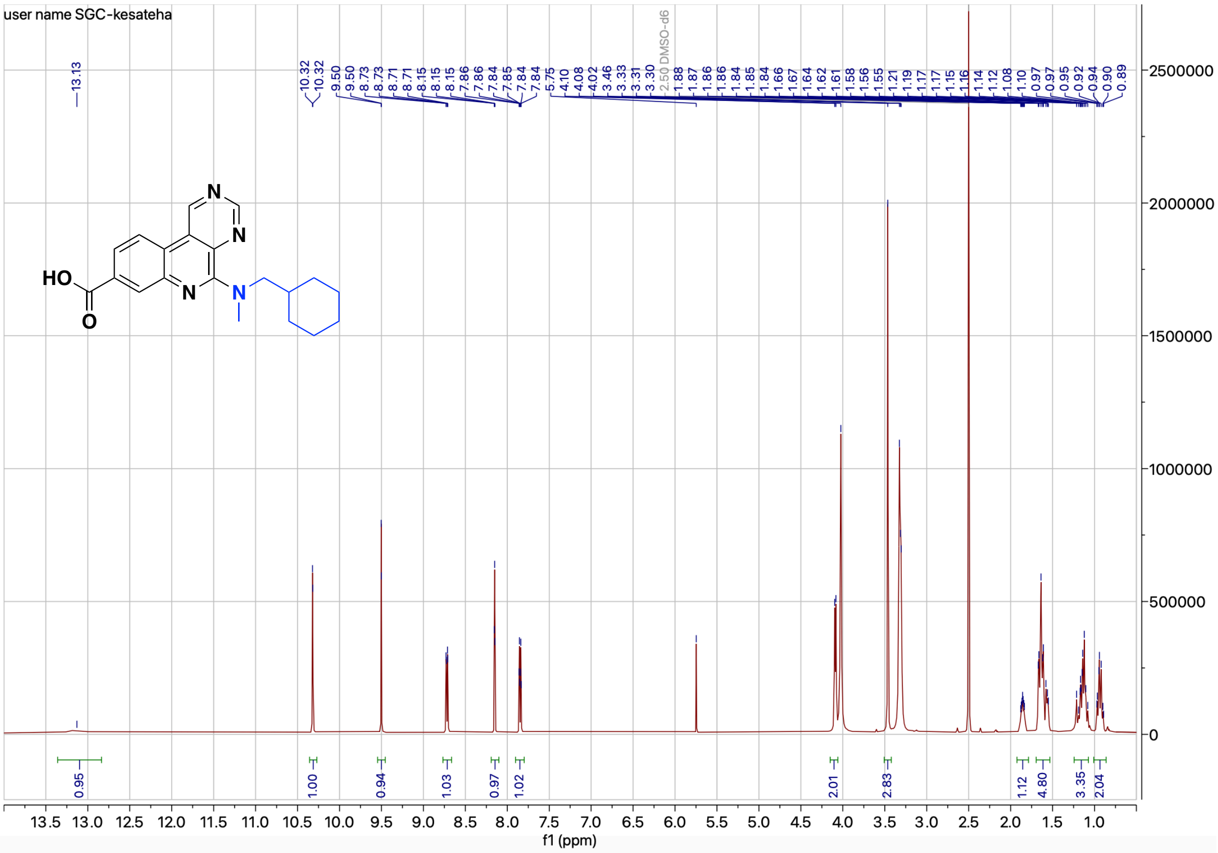

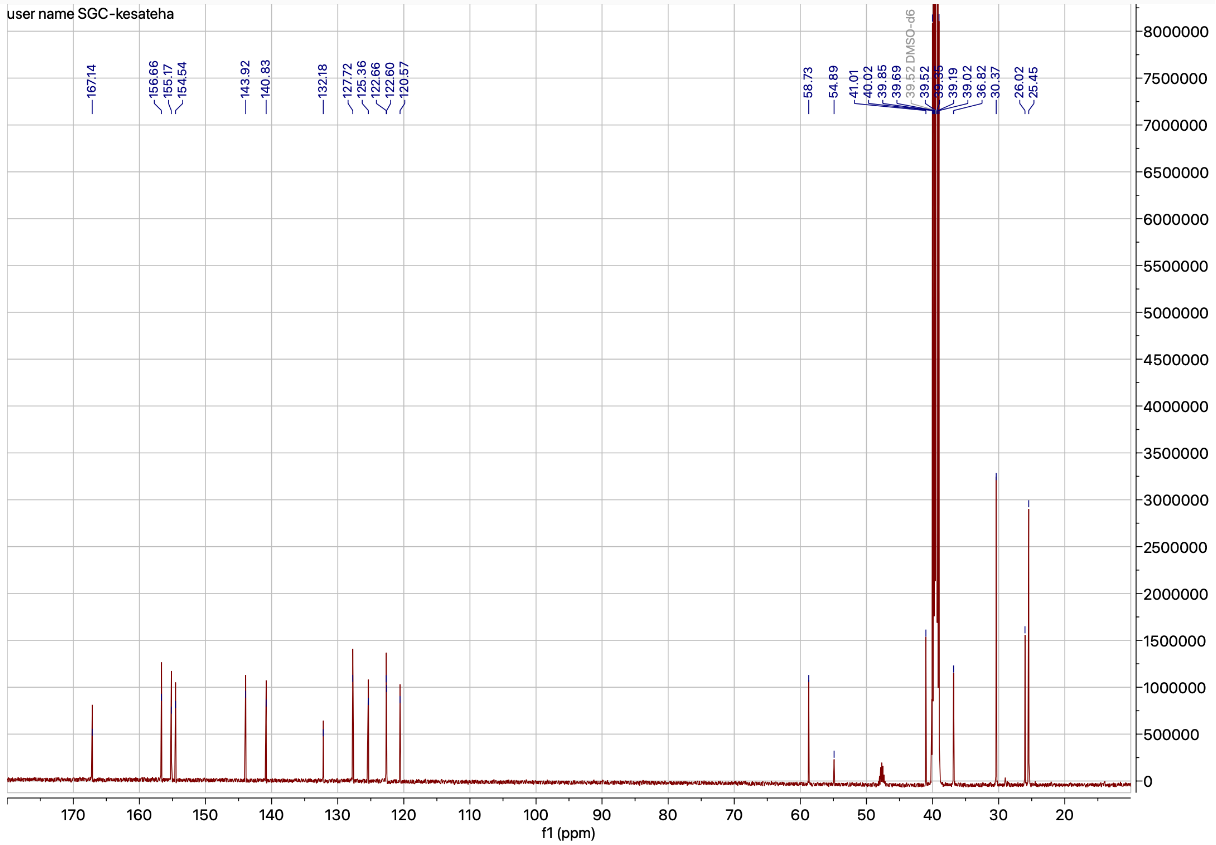


**Figure S32.** $^{1}$H and $^{13}$C NMR spectrum of compound **4ad**

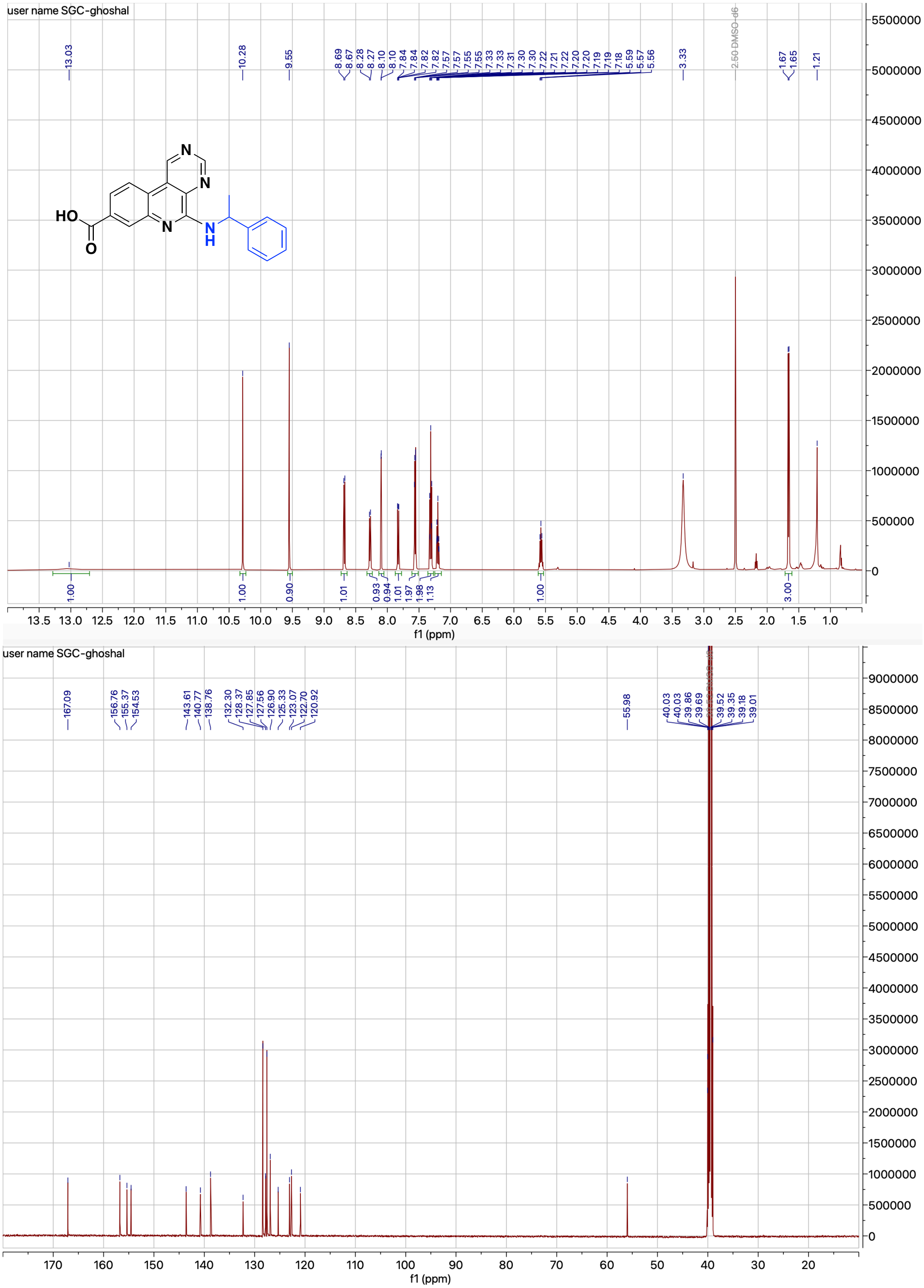


**Figure S33.** $^{1}$H and $^{13}$C NMR spectrum of compound **4ae**

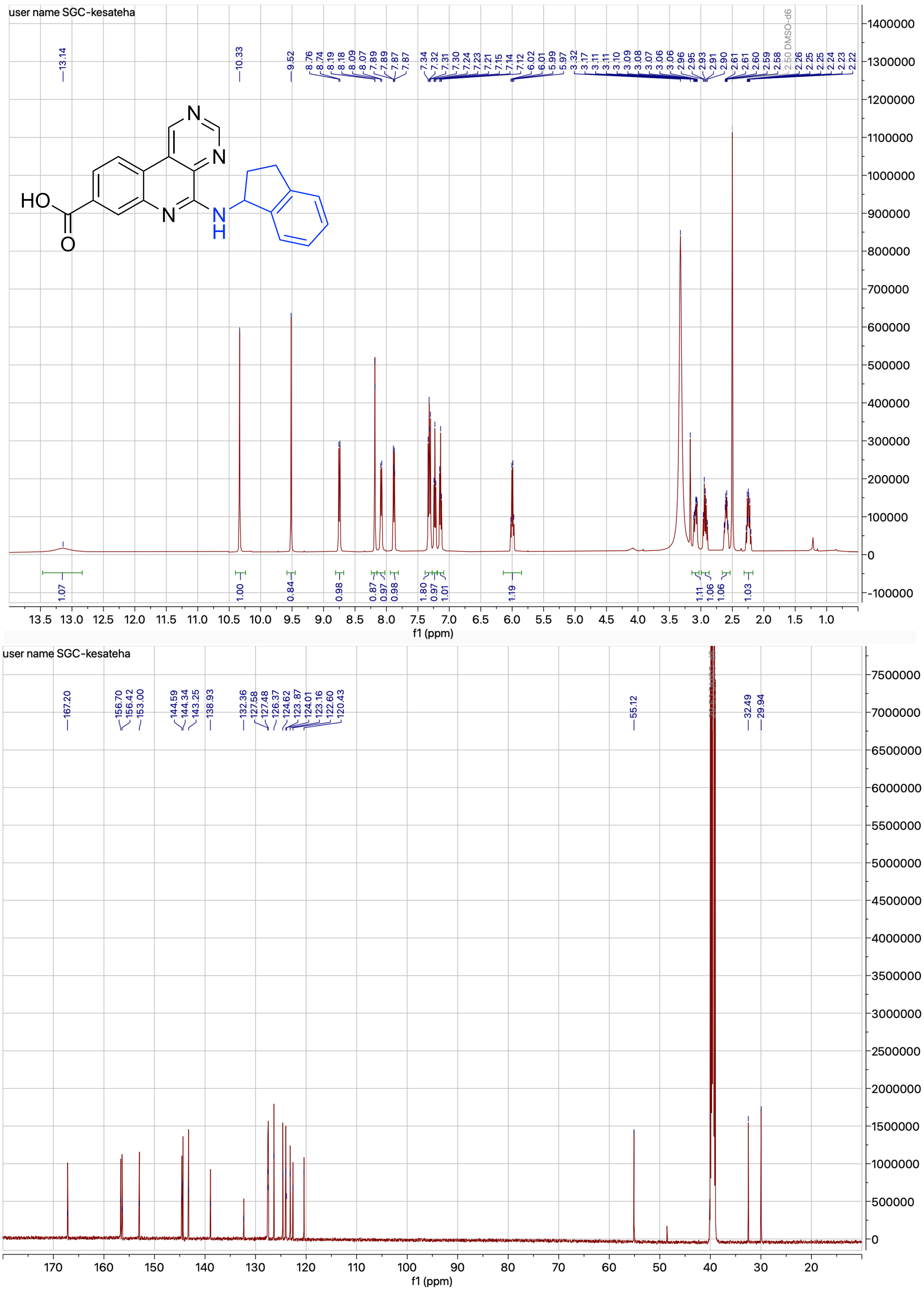


**Figure S34.** $^{1}$H and $^{13}$C NMR spectrum of compound **4af**

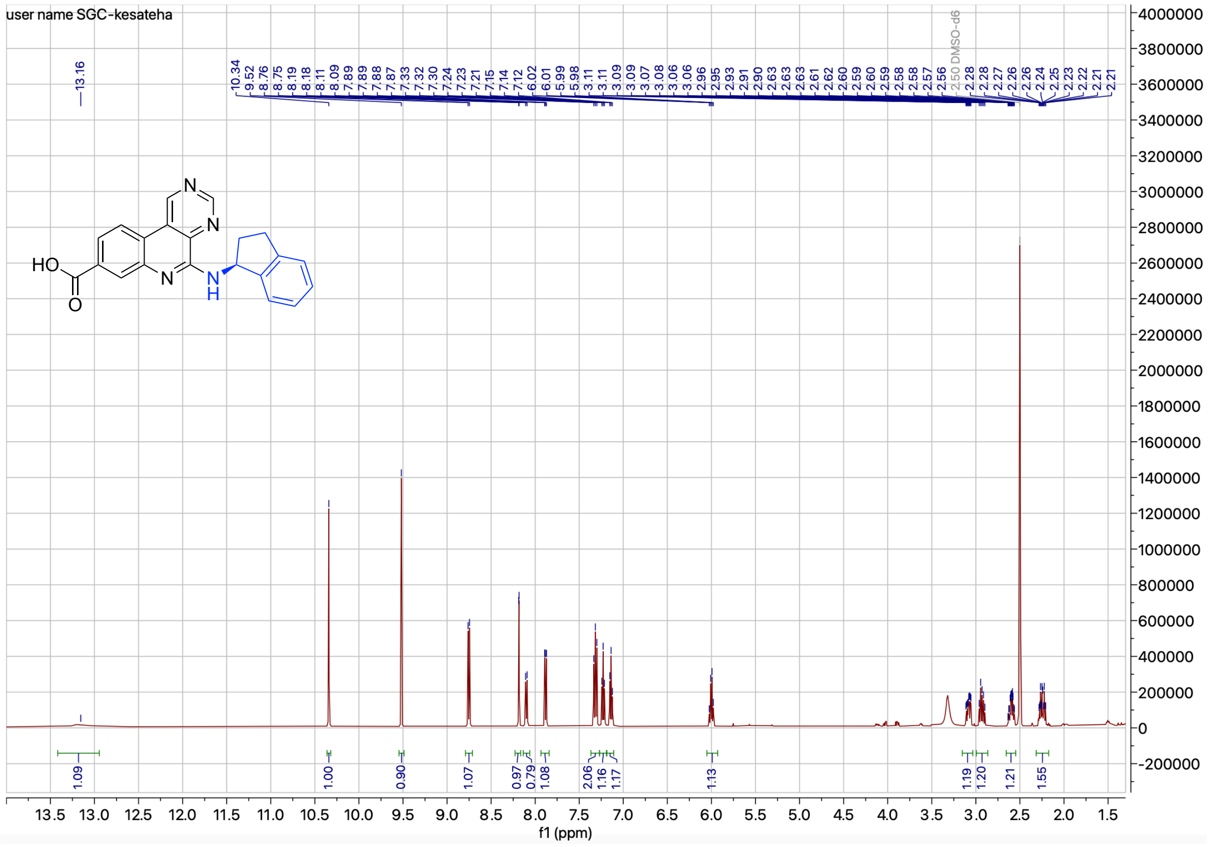


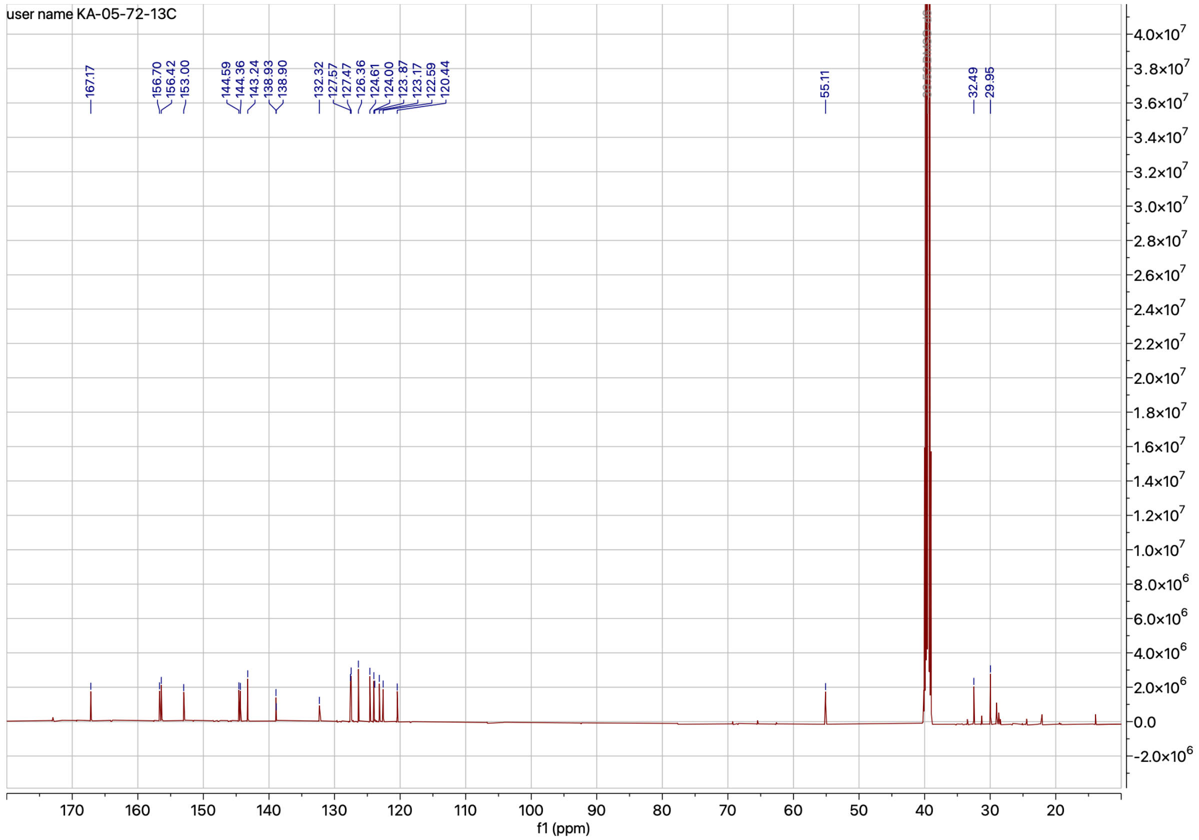


**Figure S35.** $^{1}$H and $^{13}$C NMR spectrum of compound **4ag**

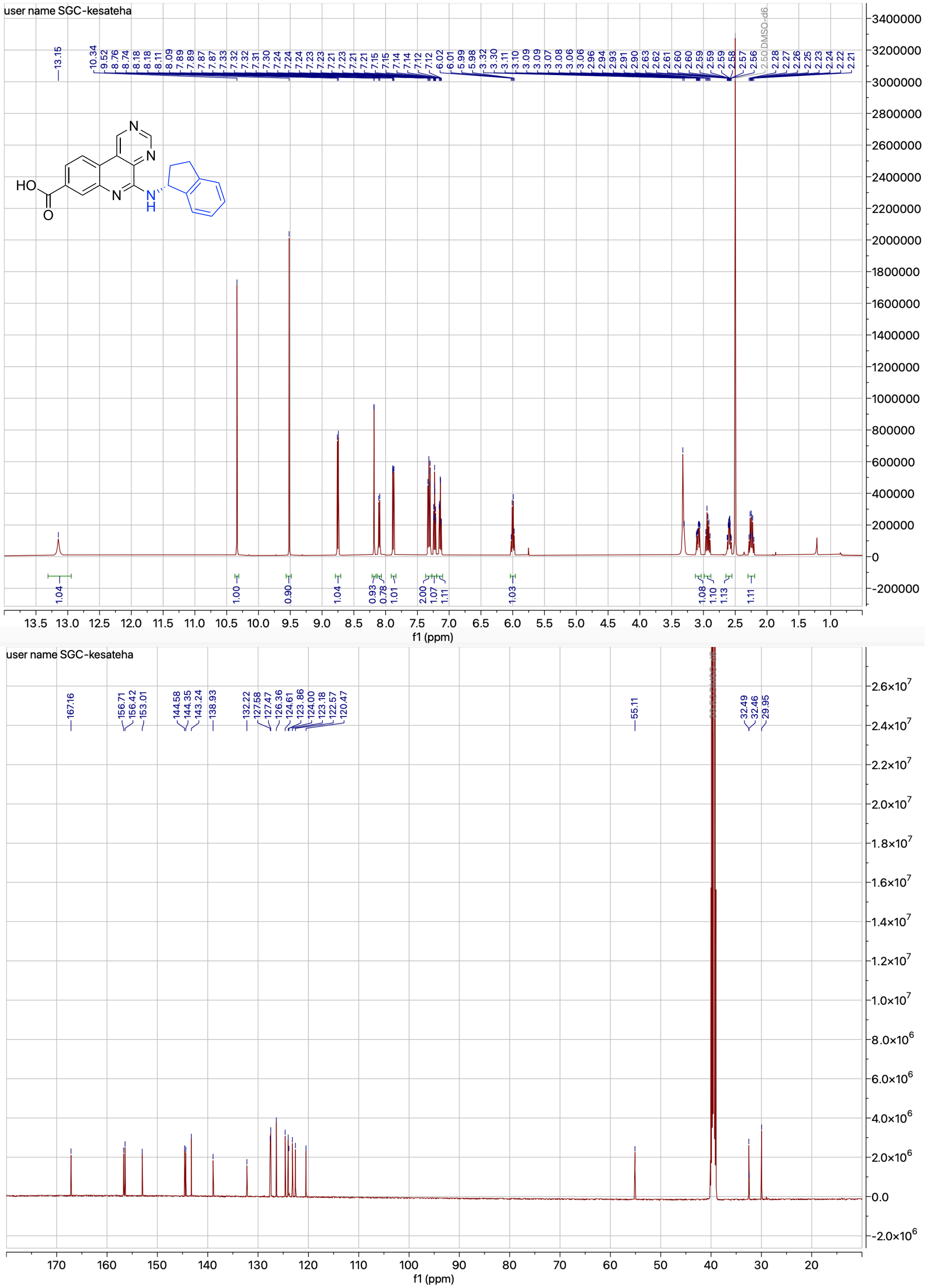


**Figure S36.** $^{1}$H and $^{13}$C NMR spectrum of compound **4ah**

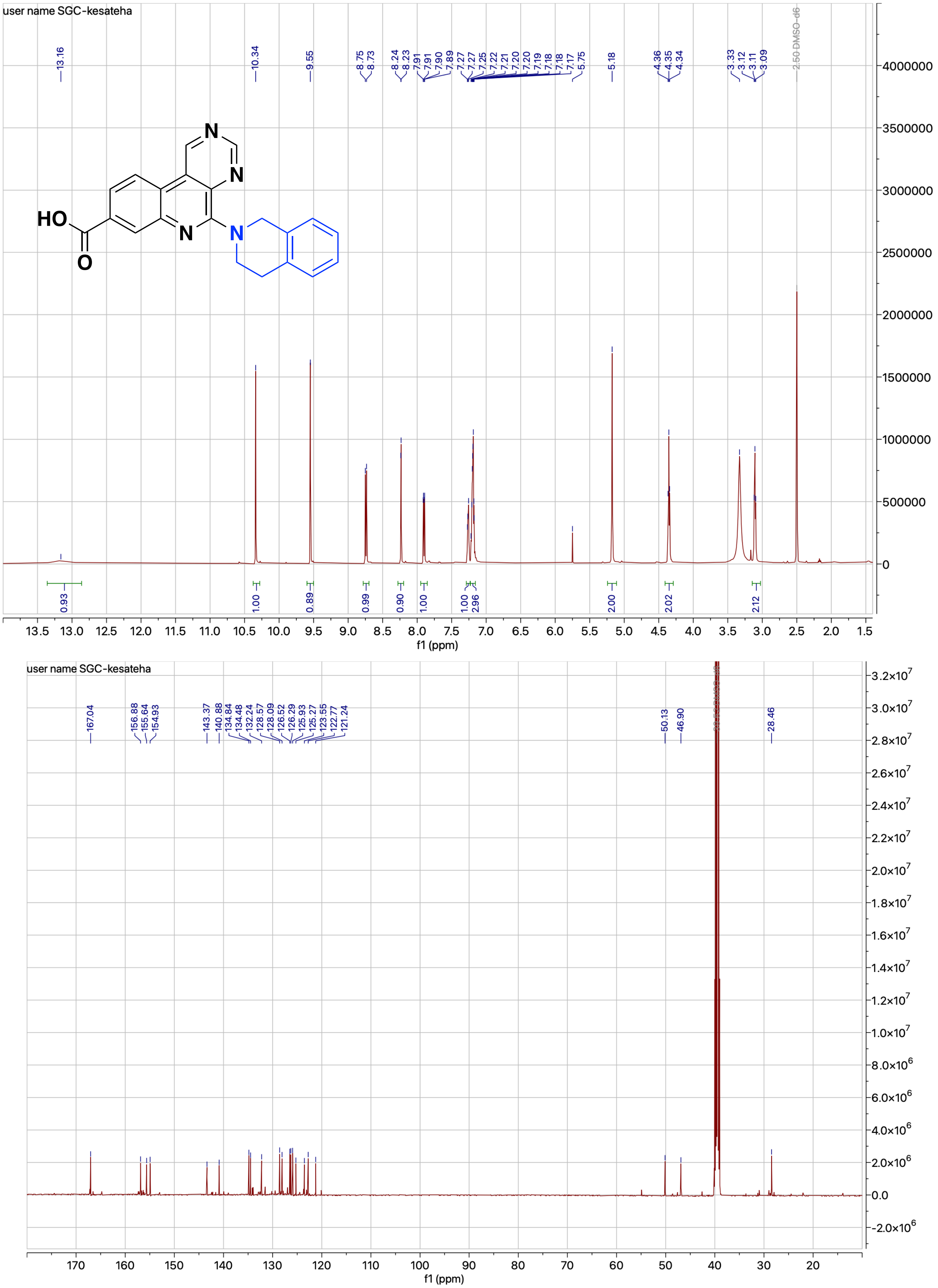


**Figure S37.** ¹H and ¹³C NMR spectrum of compound **4ai**
